# Oleanolic Acid: A Promising Antioxidant—Sources, Mechanisms of Action, Therapeutic Potential, and Enhancement of Bioactivity

**DOI:** 10.3390/antiox14050598

**Published:** 2025-05-16

**Authors:** Andrzej Günther, Barbara Bednarczyk-Cwynar

**Affiliations:** 1 Department of Organic Chemistry, Faculty of Pharmacy, Poznan University of Medical Sciences, Collegium Pharmaceuticum 2 (CP.2), Rokietnicka Str. 3, 60-806 Poznan, Poland; andrzej.gunther@me.pl; 2 Center of Innovative Pharmaceutical Technology (CITF), Rokietnicka Str. 3, 60-806 Poznan, Poland

**Keywords:** natural compounds, bioactive compounds, medicinal plants, triterpenes, oleanolic acid, antioxidant activity, anti-aging, antioxidant compounds

## Abstract

This review discusses the antioxidant potential of oleanolic acid, a triterpene compound present in many medicinal and edible plants. The authors analyze various studies that confirm numerous pharmacological properties of this compound, such as its anticancer, antidiabetic, neuroprotective, osteoprotective, anti-obesity, and anti-inflammatory effects. OA, as a natural antioxidant, plays an important role in neutralizing reactive oxygen species, which contribute to the oxidative stress that is responsible for the development of many diseases, including cancer and cardiovascular and neurodegenerative diseases. This article also presents natural sources of OA, including grapes, olives, and apples, and discusses the mechanisms of its antioxidant action, including the inhibition of lipid peroxidation and the modulation of signaling pathways related to inflammatory processes. In addition, there are research results that indicate the therapeutic benefits of OA in the treatment of diabetes and neurodegenerative diseases, as well as its potential to protect the heart, liver, and kidneys from oxidative damage. In conclusion, OA has potent antioxidant properties that can be used in the prevention and treatment of many diseases related to oxidative stress. This article also presents the possibility of increasing the bioavailability of OA through the use of nanoparticle and liposome technology.

## 1. Introduction

**Figure 1:**
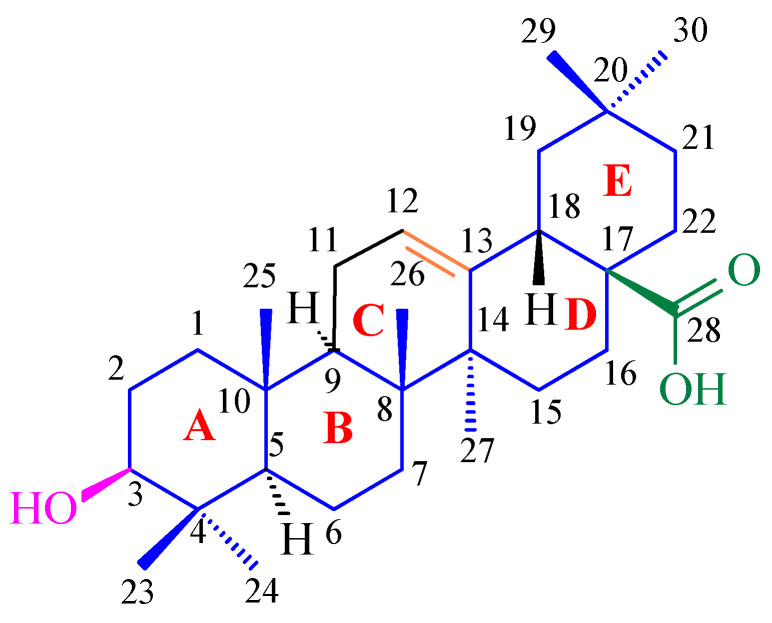
Structure of oleanolic acid. Chiral carbon atoms of OA molecule: C-3, C-5, C-8, C-9, C-10, C-14, C-17, and C-18.

**Figure 2:**
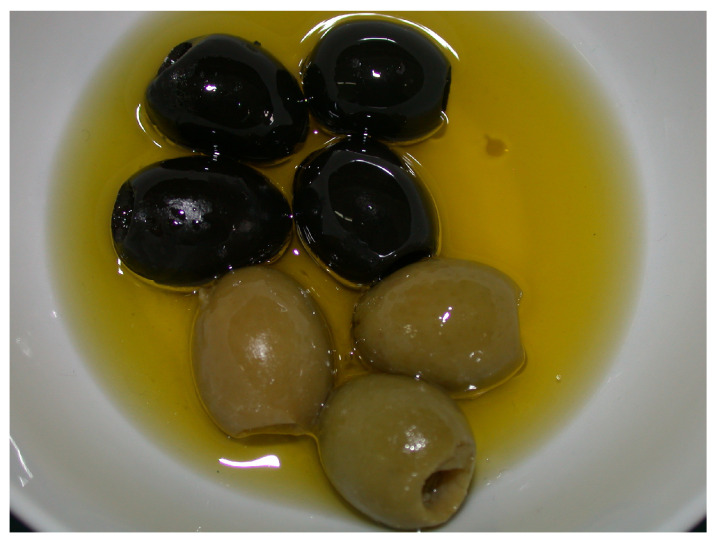
One of the sources of OA—olives (*Olea europaea* L.; Oleaceae). OA content in olive leaves: 25–35 mg/g (2.5–3.3%) of dry weight [3,4]; OA content in olive fruits (peels): 5.2 mg/g (0.52%) of dry extract [5]. Photo by B. Bednarczyk-Cwynar.

**Figure 3:**
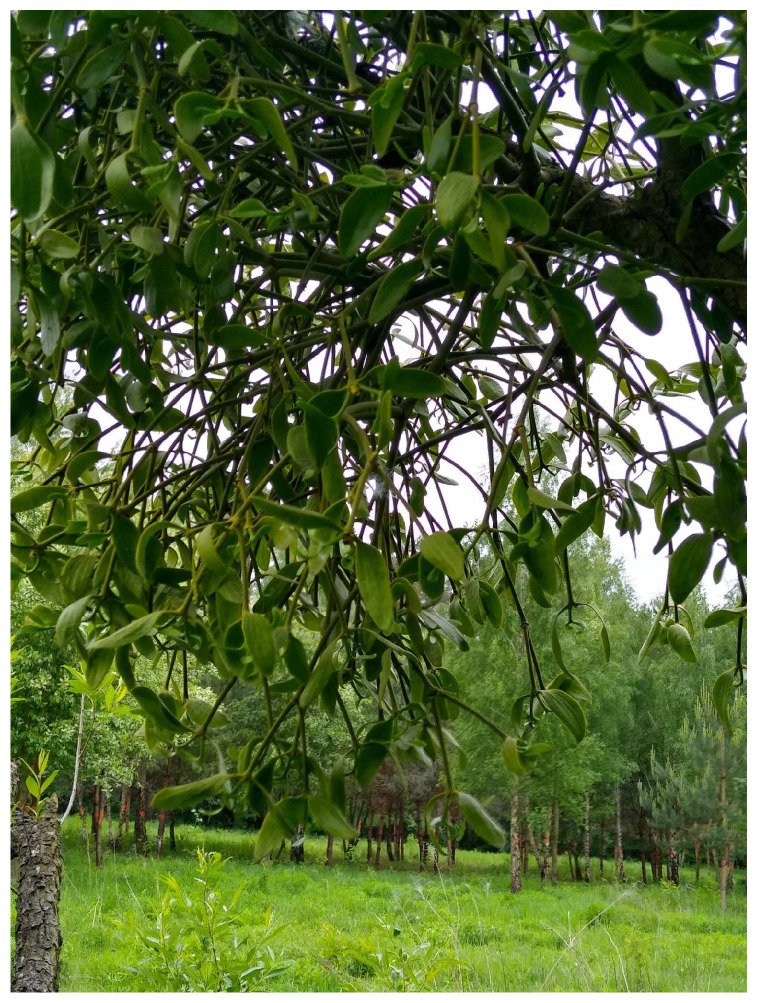
One of the sources of OA—common mistletoe (*Viscum album* L.; Santalaceae). OA content in mistletoe leaves: 8.52 mg/g [7]. Photo by B. Bednarczyk-Cwynar.

Terpenoids, also known as isoprenoids, are an extensive group of plant secondary metabolites that comprises at least 40,000 species [1]. In the plant world, the most abundant and widespread group of terpenoids are the triterpenoids, which are composed of 30 carbon atoms that form a variety of carbon skeletons. The structure of these skeletons is the basis for the division of triterpenoids into several subgroups, the most important of which are oleananes, ursanes, lupanes, and friedelanes. Of these triterpenoids, oleananes are the most common and abundant compounds. This group’s representative is oleanolic acid (common abbreviation: OA; Figure 1); the presence of this compound has been confirmed in at least 1600 species of medicinal and edible plants [2]. The richest sources of OA include the leaves and fruits of the European olive (*Olea europaea* L.; Oleaceae; Figure 2) [3,4,5], marigold herb (*Calendula officinalis* L.; Asteraceae; the wild-type of this plant and its hairy roots produce OA and its saponins) [6], and mistletoe herb (*Viscum album* L.; Santalaceae; Figure 3) [7].

OA, being a natural antioxidant, is a valuable element in the fight against the various diseases. The aim of this article is a comprehensive analysis of the research on the antioxidant potential of OA and its derivatives, as well as to present its mechanisms of action and possible clinical applications.

The biosynthesis of OA in plants is initiated by the formation of two types of isoprene units, namely isopentenyl pyrophosphate (IPP) and its isomer dimethylallyl pyrophosphate (DMAPP), which is derived from mevalonic acid (MVA) or the methylerythritol phosphate (MEP) pathway [8]. The detailed course of the biosynthesis steps that lead to 2,3-oxidosqualene is given in the literature [8]. 2,3-Oxidosqualene is a precursor of triterpenes, and the stages of biochemical transformations that lead, e.g., to OA are also detailed in the literature [9].

OA is an important substrate that is used for the synthesis of various derivatives that are predicted to have pharmacological properties. A second reason why this compound has attracted the attention of scientists is its numerous and valuable therapeutic properties, which include anti-diabetic [10], anti-obesity [11], neuroprotective [12], osteoprotective [13], anti-inflammatory [14], hepatoprotective [15], cytotoxic [16], and other properties.

Oxidative stress causes cancer, along with many other diseases such as neurodegenerative diseases, aging, cataracts, rheumatoid arthritis, cardiovascular diseases, autoimmune diseases, and others [17]. Oxidative stress arises from an overproduction of reactive oxygen species (ROS) or their improper utilization within the body [18]. While free radicals play a beneficial role in normal physiological conditions by influencing cellular responses and immune functions [17], high concentrations of free radicals can lead to oxidative stress, causing damage to cellular structures such as the deoxyribonucleic acid (DNA), lipids, and proteins [19].

## 2. Natural Sources of Antioxidant OA

As already mentioned above, OA occurs in numerous edible, medicinal, and wild plants. In these plants, OA is one of many pharmacologically active substances that influence and thus determine the pharmacological properties of extracts and other medicinal forms obtained from these plants. Below are examples of medicinal and edible plants rich in OA. Studies confirm that both pure OA and its extracts exhibit strong antioxidant effects.

For instance, common grapes (wine grapes, *Vitis vinifera* L.; Vitaceae), known for their antioxidant properties [20], are commonly used in traditional medicine for the treatment of various diseases [21]. Sasikumar and co-workers obtained a methanol extract from dried wine grapes and, next, isolated OA from the extract without using any chromatographic techniques. The purity of the isolated OA was confirmed with the application of spectral data and melting point analysis [21]. Then, the antioxidant activity of the obtained OA was examined using the 1,1-diphenyl-2-picrylhydrazyl (DPPH) method. The results showed that OA has significant activity in neutralizing free radicals: the radical scavenging activity (RSA) was 88.30%, as reported in the original study [21]. This value was comparable to those of antioxidants such as ascorbic acid and gallic acid. The minimum inhibitory concentration (IC_50_) value was 61.5 µg/mL. Moreover, the anticancer effect of OA was tested on the HCT-116 (colon cancer) cell line. OA reduced the cell viability in a concentration-dependent manner, with an IC_50_ value of 40 µg/mL after 48 h. In the study, OA contained in black raisin showed evidence of its antiproliferative capacity in the tests carried out on the HCT-116 cell line, especially in the treatment of colorectal cancer, due to its antioxidant and cytotoxic properties against cancer cells. The article highlights the need for further research to understand the molecular mechanisms behind the anticancer effects of OA and its potential use in the development of herbal medicines for cancer treatment [21].

The traditional Mediterranean diet is characterized by the consumption of foods such as grapes, wine, raisins, olives, and virgin olive oil [22]. All the plant raw materials mentioned above contain OA as one of their main antioxidant substances [22]. According to the research conducted by Sánchez-Quesada and co-workers [22], OA that was present in the skins of fruits such as olives (*Olea europaea* L.; Oleaceae; Figure 3) and wine grapes (*Vitis vinifera* L.; Vitaceae) showed an effect against oxidative stress in a human breast epithelial cell line (MCF-10A), reducing the level of ROS in the cells. However, in highly invasive cancer cells (MDA-MB-231, triple negative human epithelial breast cancer cells), OA increased the ROS levels, which could lead to cancer cell death.

In their investigation, Sánchez-Quesada and co-workers presented that OA demonstrated a protective effect upon MCF10A cells by reducing their basal ROS level. Upon the induction of oxidative stress, the OA maintained this protective effect, decreasing the cellular sensitivity to ROS. Since ROS can initiate carcinogenesis by inducing permanent DNA damage, OA may function as an antioxidant. By mitigating oxidative damage, OA could help protect cells in microenvironments that are prone to oxidative stress and which may otherwise favor tumor development [22].

In MDA-MB-231 cells, OA exhibited a pro-oxidative effect. At basal conditions, lower concentrations of OA appeared to elevate the intracellular oxidative stress. Furthermore, when oxidative stress was induced with hydrogen peroxide (H_2_O_2_), OA significantly enhanced the cells’ ROS levels—by approximately 30% compared to the control. These findings suggest that OA may act as a pro-oxidant in highly invasive breast cancer cells (such as MDA-MB-231). This effect could be therapeutically relevant, as elevated ROS levels have the potential to inhibit carcinogenesis and trigger apoptosis in tumor cells [22].

Thus, OA may play a dual role: such differential activity may make OA a valuable compound in the development of breast cancer therapies, especially in the treatment of more aggressive forms [22].

Yunoki and co-workers tested the concentration of OA in pomace, which was a byproduct of the production of wine from different cultivars of Kiyomi oranges (e.g., *Citrus reticulata* Blanco × *Citrus* × *aurantium* L.; Rutaceae) [23].

**Figure 4:**
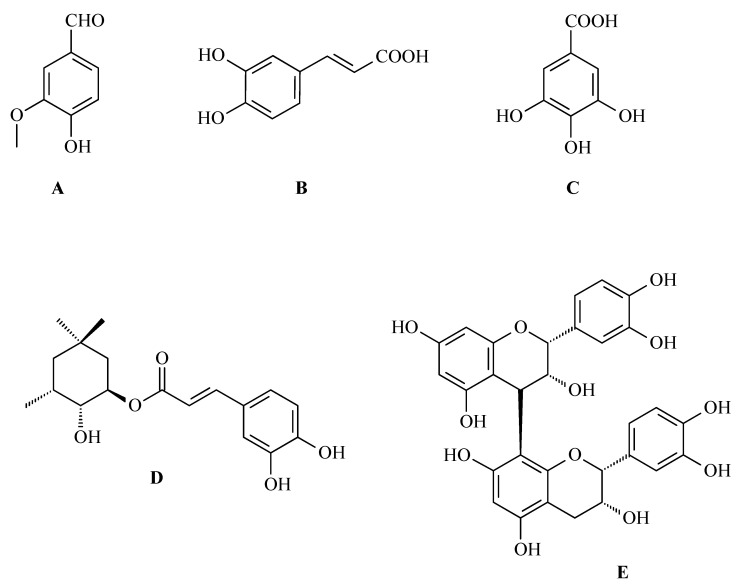
Main fenolic components of MPE: (**A**) = vanilic aldehyde; (**B**) = caffeic acid; (**C**) = gallic acid; (**D**) = chlorogenic acid; (**E**) = protocyanidin B2. Compound contents in MPE: vanilic aledyde: 0.0005 mg/g, caffeic acid: 0.0114 mg/g, gallic acid: 0.0007 mg/g, chlorogenic acid: 0.0198 mg/g, procyanidin B2: 0.0125 mg/g [24].

Bai and co-workers, for the first time, evaluated the antioxidant activities of mango (*Mangifera indica* L.; Anacardiaceae) peel extract (MPE) using in vitro tests. This extract was evaluated in the context of its potential applications in the fight against lung cancer [24]. The study analyzed the chemical composition of MPE and its antioxidant and cytotoxic effects on A549 cancer cells (lung cancer line). High-performance liquid chromatography (HPLC) analysis identified the main phenolic compounds in the extract, which included vanilic aldehyde (Figure 4A), caffeic acid (Figure 4B), gallic acid (Figure 4C), chlorogenic acid (Figure 4D), procyanidin B2 (Figure 4E), and OA (Figure 1). The extract showed strong antioxidant properties, with a 92 ± 4.2% DPPH radical scavenging rate and a 79 ± 2.5% of 2,2′-azino-bis-3-ethylbenzthiazoline-6-sulfonic acid (ABTS) radical inhibition rate. Among the phenols that were tested, shown in Figure 4A–E, gallic acid showed the strongest antioxidant properties [24].

In the context of anticancer research, MPE had moderate cytotoxic effects, but OA proved to be the most effective, with an IC_50_ value of 4.7 µmol. This IC_50_ value for OA was comparable to the IC_50_ value of the popular anticancer drug 5-fluorouracil (3.5 µmol). Thus, mango shells are a valuable source of bioactive compounds that can be used in the production of functional foods and in the treatment of free radical-related diseases and cancer, especially lung cancer [24].

**Figure 5:**
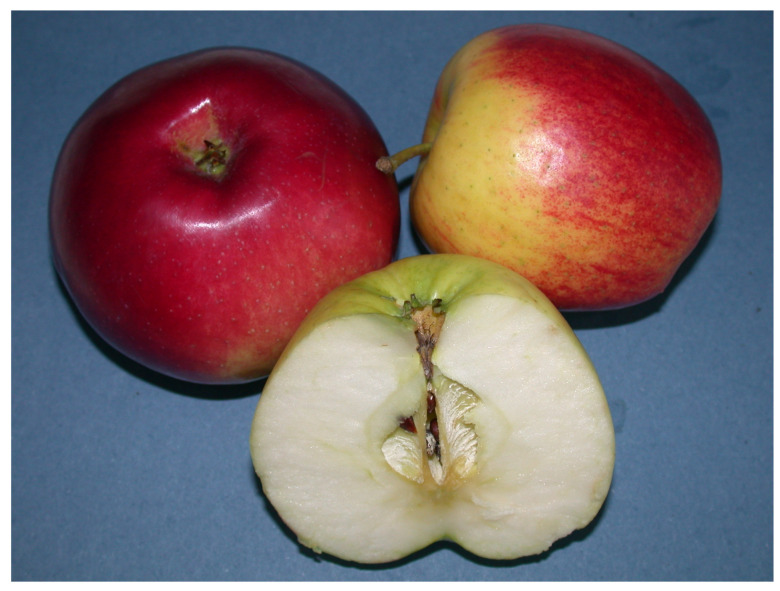
One of the sources of OA—apples (*Malus domestica* (Suckow) Borkh; Rosaceae). OA content in apples peels: 3.6 mg/g dry peel weight [26]. Photo by B. Bednarczyk-Cwynar.

**Figure 6:**
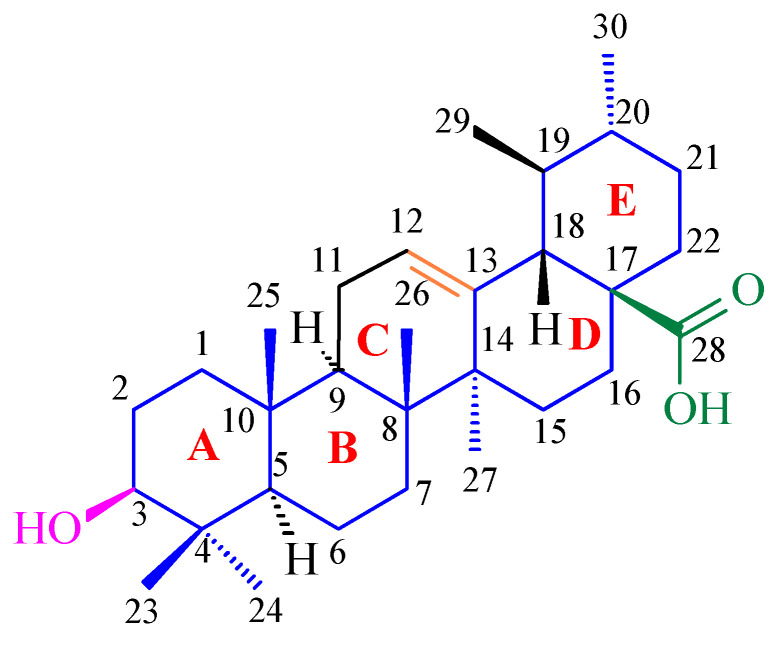
Structure of ursolic acid (UA). Chiral carbon atoms of OA molecule: C-3, C-5, C-8, C-9, C-10, C-14, C-17, C-18, C-19, and C-20.

Apples (*Malus domestica* (Suckow) Borkh.; Rosaceae; Figure 5) are rich sources of UA (Figure 6) and OA, which are the primary triterpenes found in apple peel [25]. OA (Figure 1) and UA (Figure 6) are isomers and differ in the position of one substituent in their E rings.

**Figure 7:**
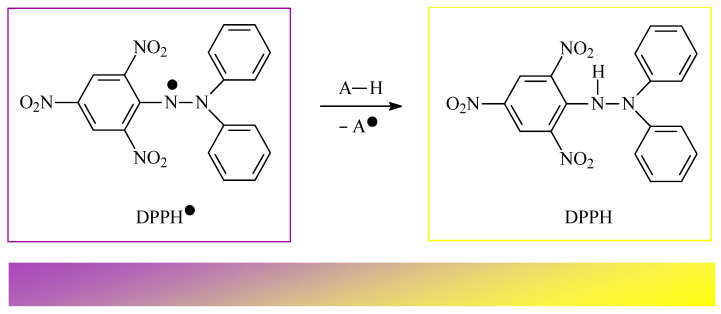
Schematic representation of how the DPPH test works. The assay is based on the reduction of the stable, violet-colored DPPH radical (DPPH^•^), which exhibits a characteristic absorbance at 515–517 nm. Upon interaction with an antioxidant (A–H = electron or proton donor), the DPPH^•^ is reduced, resulting in a color change from violet to yellow (DPPH), which reflects the radical-scavenging capacity of the tested compound.

Odun-Ayo and co-workers determined the content of both abovementioned acids in the methanol extract of various apple peel cultivars and then applied a DPPH assay to determine the antioxidant potential of the apple peel extracts [25]. Apples, particularly their peels, are a rich source of triterpenes, which may reduce the risk of diseases such as cancer, cardiovascular disease, and diabetes [25]. The study included three apple varieties: Red Delicious (RD), Royal Gala (RG), and Granny Smith (GS). The content of UA and OA was analyzed using the HPLC method. For quantitative analysis, the concentrations of UA and OA in the apple peel extract were determined using standard curves prepared from known concentrations of each triterpene acid. The ability of OA and UA to neutralize free radicals was tested with the application of a DPPH assay. A schematic representation of how the DPPH test works is presented in Figure 7. The results showed that the Red Delicious variety had the highest UA and OA content (248.02 ± 0.08 μg/mL and 110.00 ± 0.08 μg/mL, respectively) and the highest antioxidant capacity: 97.3 ± 0.40%, as reported in the original paper [25]. In turn, the apples of the Granny Smith variety were characterized by the lowest results. A strong positive correlation was found between the acid content and the apples’ antioxidant activity, particularly in the case of the Red Delicious variety. The results indicated a concentration-dependent increase in antioxidant capacity for both UA and OA, confirming their potential as effective antioxidants. The study confirmed that RD and RG apple peels are a valuable source of triterpenes, making them beneficial both when included in humans’ diet and in the production of supplements that can support human health [25].

Essential oils and extracts of white-leaved catmint (*Nepeta leucophylla* Benth.; Lamiaceae), a wild aromatic herb mainly found in the Western Himalayas, have been reported to display antibacterial, antioxidant, and antifungal activities [27]. Sharma and co-workers tested compounds isolated from catmint extract for all of the above activities. The results revealed that OA is indeed a less active antioxidant agent than, for example, ascorbic acid, but much stronger than, for example, squalene, from which, as a result of multi-stage enzymatic biosynthesis, all triterpenes are produced [27].

Only a few examples of studies on the antioxidant activities of plant extracts that contain OA as their main active ingredient are discussed in more detail above. Many more studies on the same topic exist in the scientific literature. For example, Assimopoulou and co-workers studied the possible antioxidant effects of natural resins such as the resin of the mastic tree (*Pistacia lentiscus* L.; Anacardiaceae), the resin of Indian frankincense (*Boswellia serrata* Roxb. ex Colebr.; Burseraceae), and the resin of common myrrh (*Commiphora myrrh* Nees; Burseraceae), as well as the use of OA and UA as antioxidant triterpenes. In their experiments, they have also used lard and some vegetable oils, such as olive, corn, and sunflower oils. Lard and these oils were applied as oil bases and dispersion media for resins and triterpenes in the presented experiments [28].

**Figure 8:**
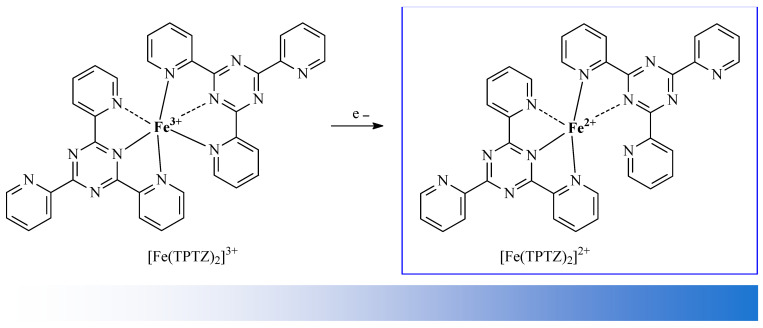
Schematic representation of how the FRAP test works. The assay is based on the reduction of the stable, colorless ferric-tripyridyltriazine, [Fe(TPTZ)_2_]^3^^+^, which exhibits a characteristic absorbance at 593 nm. Upon interaction with an antioxidant (e^−^, electron donor), [Fe(TPTZ)_2_]^3^^+^ is reduced, resulting in a color change to blue, which is directly proportional to the antioxidant power of the tested compound.

The antioxidant activity of extracts prepared from whole fruits, as well as from the skins and seeds of the fruits of fox grapes (*Vitis labrusca* L.; Vitaceae), were tested by Koca and co-workers [29]. Their research shows that OA was one of the main pharmacologically active substances in the studied extracts. The antioxidant activity of the extracts was evaluated with DPPH and ferric reducing antioxidant power ability (FRAP; Figure 8) assays [29]. Siddiqui and co-workers tested 15 fractions of extracts prepared from hibiscuses (*Hibiscus calyphyllus* Cav.; Malvaceae, *H. deflersii* Schweinf. ex Cufod.; Malvaceae, and *H. micranthus* L.f.; Malvaceae). To assess the level of antioxidant activity of the mentioned extracts, the scientists applied the DPPH assay. The main biologically active compound in these extracts was also found to be OA [30]. Adjei and co-workers evaluated methanolic extracts of black plum (*Vitex doniana* Sweet; Vitaceae) fruits that contained OA as a main active compound with the application of a DPPH assay [31]. Biltekin and co-workers tested the antioxidant activity of hexane extracts obtained from olive fruit (*Olea europaea* L.; Oleaceae) pomace. These extracts exhibited strong antioxidant activity in DPPH and FRAP methods, mainly due to the presence of one of the most important chemical components—OA [5]. Reddy and co-workers tested the antioxidant and anti-hypertensive properties of the methanol extracts of six plants and some compounds isolated from these plants. The plants used in their tests were mugwort (African wormwood; *Artemisia afra* Jacq. Ex Willd.; Asteraceae), horsewood tree (*Clausena anisata* (Willd.) Hook.f. ex. Benth; Rutaceae), dikbas (South African wild pear; *Dombeya rotundifolia* (Hochst.) Planch.; Malvaceae), morula (cider tree; *Sclerocarya birrea* (A.Rich.) Hochst.; Anacardiaceae), red currant tree (*Searsia chirindensis* (Baker f.) Moffett; Anacardiaceae), and pepper-bark tree (*Warburgia salutaris* (Bertol.f.) Chiov.; Canellaceae). The antioxidant activity of the studied extracts and compounds was determined using the DPPH free radical scavenging assay and the nitric oxide (NO) scavenging assay [32]. The presence of OA in, e.g., lemon balm extract (*Melissa officinalis* L.; Lamiaceae) [33], henna seeds (*Lawsonia inermis* L.; Lythraceae) [34], fruits of Chinese jujube (*Ziziphus jujuba* Mill.; Rhamnaceae) [35], aerial parts of grape-scented sage (*Salvia melissiflora* Benth.; Lamiaceae) [36], and other medicinal and edible plants has also been tested. Numerous studies are being conducted around the world on the mechanisms of the antioxidant activity of OA.

Table 1 presents the OA content in the plants mentioned in the presented publications [5,21,22,23,24,25,26,27,28,29,30,31,32,33,34,35,36]. The OA content was converted to mg/g (or g/mL) of plant material (or plant extract) and expressed as a percentage.

Table 2 presents the antioxidant activity of OA as determined by the application of the various methods used in the cited papers [5,21,22,23,24,25,26,27,28,29,30,31,32,33,34,35,36].

## 3. Research on the Mechanisms of OA Antioxidant Activity

Research on the mechanisms of the antioxidant action of OA focuses on its ability to inhibit lipid peroxidation processes, neutralize ROS, and increase the activity of antioxidant enzymes [37]. OA also can modulate signaling pathways related to inflammatory processes, which suggests that it has a protective effect in the context of chronic diseases such as diabetes and neurodegenerative diseases.

In their research, Wang and co-authors [37] conducted a number of studies assessing the probability of various potential mechanisms of the antioxidant activity of OA. The ability of OA to exert the following forms of antioxidant activity was tested: the deactivation of free radicals, the protection of cell membranes, the exertion of an impact on the redox balance (i.e., on the regeneration of endogenous antioxidants such as glutathione and GSH), the induction of antioxidant enzymes (i.e., the activation of transcription factors which regulate the expression of antioxidant enzymes), and the inhibition of pro-inflammatory pathways.

**Figure 9:**
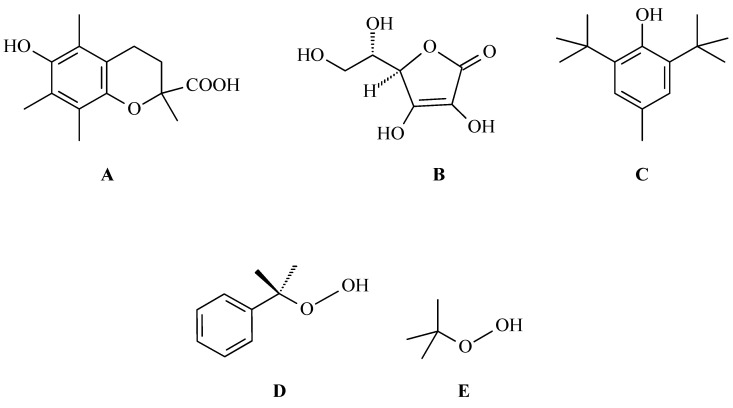
Free radical scavengers: (**A**): trolox, (**B**): vitamin C, (**C**): butylated hydroxytoluene (BHT). Free radicals producers: (**D**): cumine hydroperoxide, (**E**): t-butyl hydroperoxide (tBHP).

To assess the ability of OA to deactivate free radicals, Wang and co-authors used the DPPH, cobalt(II) ion, and ethylenediaminetetraacetic acid-induced (Co(II)/EDTA-induced) chemiluminescence and peroxide anion radical assays [37]. In the first one, the DPPH assay, the ability of OA to scavenge DPPH radicals was tested to assess whether the mentioned triterpene has the ability to donate electrons or hydrogen atoms. In the second test, it was assessed whether OA has the ability to scavenge hydroxyl radicals. The results showed that OA, compared to antioxidants such as trolox (Figure 9A), vitamin C (Figure 9B), and butylated hydroxytoluene (BHT; correct name: 4-methyl-2,6-di-*tert*-butyl phenol; Figure 9C), exhibits a limited ability to scavenge DPPH radicals; in the second test, the antioxidant activity was moderate. Much better results were obtained for OA in the third test, which revealed that OA has the ability to scavenge peroxide anion radicals at a level comparable to that of vitamin E, which is a known and effective antioxidant.

The second aspect of interest to scientists was the ability of OA to inhibit lipid peroxidation. Membrane lipids are particularly vulnerable to oxidation because of their high content of polyunsaturated fatty acids and their close association with both enzymatic and non-enzymatic systems within the cell membrane, which can generate free radical species [37]. The aim of these studies was to assess the ability of OA to protect cell membranes against damage caused by various factors that induce lipid peroxidation, such as ascorbate/Fe^2^^+^, cumine hydroperoxide (correct name: cumene hydroperoxide, CHP; Figure 9D), carbon tetrachloride/nicotinamide adenine dinucleotide phosphate (CCl_4_/NADPH), and *tert*-butyl hydroperoxide (tBHP; Figure 9E). The free radical-scavenging activities of OA were measured with the application of the DPPH and induced luminol chemiluminescence methods. The results, expressed as the percentage inhibition ratio, showed that OA effectively inhibited lipid peroxidation, especially in the CHP-induced model. In the ascorbate/Fe^2^^+^-induced system, OA presented moderate inhibitory effect, while in the CCl_4_/NADPH-induced system, OA showed comparable effectiveness to vitamin E. In turn, using t-butyl hydroperoxide (tBHP) to induce oxidative damage in a human fetal liver cell line (QZG cells, immortalized but not derived from cancer cells), it was shown that OA not only improved the integrity of cell membranes and reduced the generation of ROS, but also significantly reduced lactate dehydrogenase (LDH) release in a dose-dependent manner, which confirmed its protective effect against the harmful effects of ROS on cell membranes [37].

The third mechanism of the antioxidant activity of OA that was studied by Wang and co-authors [37] is based on the influence of the tested triterpene on the redox balance, more precisely on the regeneration of endogenous antioxidants, such as GSH. The results showed that OA promoted an increase in the GSH/GSSG (reduced to oxidized glutathione) ratio, indicating its role in maintaining cellular redox balance.

According to the research conducted by Wang and co-workers [37], OA has the ability to activate transcription factors which regulate the expression of antioxidant enzymes. This is another mechanism of the antioxidant activity of the discussed triterpene. Scientists have studied the expression of a key transcription factor that regulates the antioxidant response. This key transcription factor is nuclear erythroid 2-related transcription factor (Nrf2). The t-butyl hydroperoxide test showed that tBHP significantly decreased the expression of Nrf2, while OA dose-dependently increased its level. Furthermore, OA increased the expression of catalase (CAT) and peroxidoxin 1 (PRX1), key hydrogen peroxide elimination enzymes [37].

The last type of OA antioxidant mechanism, studied by Wang and co-author [37], concerned the inhibition of pro-inflammatory pathways. In a mitogen-activated protein kinase (MAPK, MAP-kinase) activation study, Wang and his group observed that OA increased the phosphorylation of cellular Jun N-terminal kinase (JNK kinase) and extracellular signal-regulated kinase (ERK kinase) but did not significantly affect p38 MAP-kinase. Three main pathways, namely the NF-κB (nuclear factor kappa B), MAPK, and JAK-STAT (Janus kinase/signal transducers and activators of transcription) pathways, are crucial in inflammation, and any dysregulation in one or more of these pathways can lead to inflammation-associated diseases. The JNK pathway, a subgroup of the MAP kinases, plays a significant role in various inflammatory disease states [38]. The ERK, another member of the mitogen-activated protein kinase family, is critical in the dysregulation of chondrocyte gene expression during chronic inflammation [39]. The results obtained by Wang et al. suggested that the JNK and ERK pathways are important in the mechanism of action of OA against oxidative stress.

The obtained results indicate that OA acts mainly through indirect mechanisms, such as increasing the level of intracellular antioxidants and activating signaling pathways that are related to MAP kinases and the Nrf2 transcription factor.

**Figure 10:**
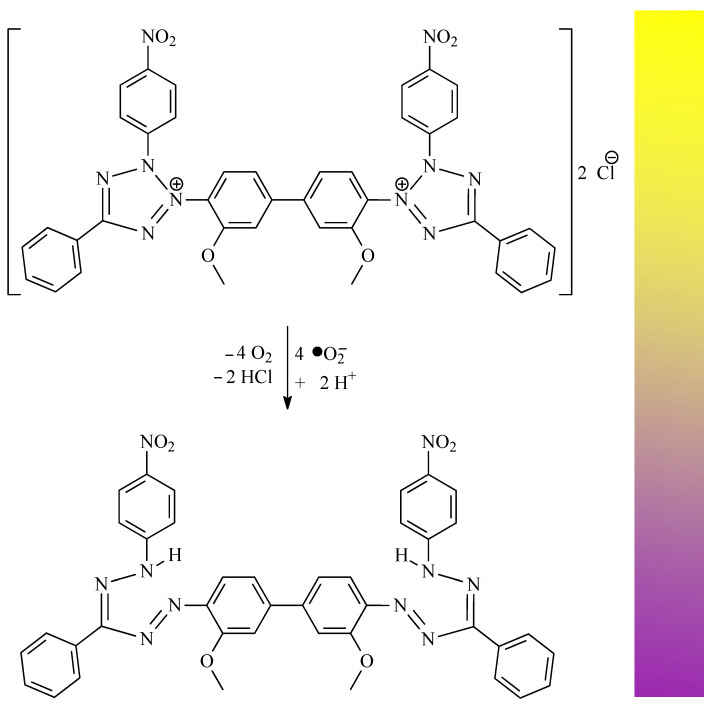
Schematic representation of how the NBT test works. The assay is based on the reduction of the stable, yellow nitroblue tetrazolium salt (NBT), which exhibits a characteristic absorbance at 385 nm. Upon interaction with superoxide radicals (^•^O_2_^−^), NBT is reduced, resulting in a color change to formazan purple, which is directly proportional to the antioxidant power of the tested compound.

Senthilkumar et al. discovered that OA significantly inhibits DPPH activity in a dose-dependent manner, with an IC_50_ value of 32.5 µg/mL [40]. Furthermore, OA exhibited superoxide anion radical scavenging activity in a nitroblue tetrazolium salt (NBT, Figure 10) assay, exhibited hydroxyl radical scavenging activity in a Fenton reaction, and has also demonstrated nitric oxide radical scavenging activity and hydrogen peroxide scavenging activity. The results of the abovementioned tests proved one of the most important mechanisms of the antioxidant activity of OA, i.e., the ability of this compound to deactivate free radicals. Senthilkumar et al. also proved the chelating activity that OA exerts on ferrous ions Fe^2+^ and the Fe^3+^ reducing power of OA. They conducted a test which showed that the formation of the ferrozine–Fe^2+^ complex is disrupted in the presence of OA and indicated that it exerts chelating activity with an IC_50_ of 0.241 mg/mL. Ferrozine forms complexes with ferrous ions, but this complex formation is interrupted in the presence of chelating agents. Ferrous ions initiate lipid peroxidation through the Fenton reaction and accelerate the formation of peroxyl and alkoxyl radicals.

Research on the antioxidant activity of OA and the mechanisms of this activity inspired scientists to conduct further studies. In 2014, Yang and his group presented the results of electrochemical and antioxidant studies of OA that were carried out using a screen-printed electrode modified with multi-walled carbon nanotubes (MWCNTs/SPE) [41]. Electrochemical analysis enabled a detailed characterization of the redox mechanisms of OA, and these results may have important implications in understanding the mechanisms of action of this compound in the context of its potential pharmacological applications. Studies have shown that the electrochemical activity of OA is strongly dependent on the pH value (the negative decadic logarithm of the proton activity in the solution under scrutiny) and the type of buffer electrolyte that are applied [41]. The pK_a_ value (the negative decadic logarithm of the acid dissociation constant) for OA is 2.52 [42]. The highest redox activity of OA was obtained in citrate buffer at pH 4.0, suggesting that this pH range favors the most stable redox reaction conditions [41].

**Figure 11:**
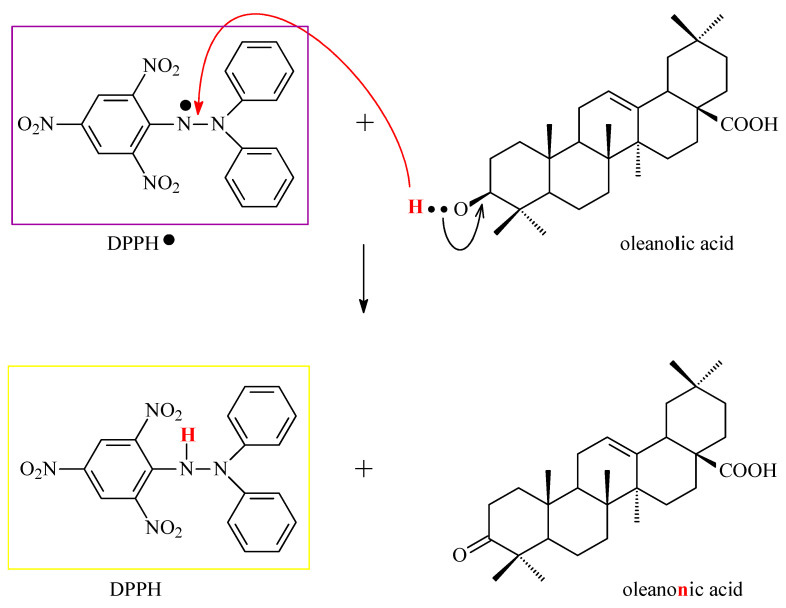
The mechanism of the antioxidant activity of OA proven with the application of DPPH assay. One equivalent of DPPH^•^ reacts with one equivalent of OA, forming an oxo derivative of OA (oleanonic acid).

Using the spectrophotometric method, the ability of OA to scavenge DPPH free radicals was examined. The results show that OA effectively neutralizes free radicals, achieving the maximum neutralization efficiency (64.3%) at concentrations above 1.0 mg/mL. The IC_50_ of OA was calculated to be 1.21 mmol for an initial DPPH concentration of 2.51 mmol, indicating a stoichiometric OA/DPPH ratio of approximately 1:1. This stoichiometric ratio of OA to DPPH suggests that one proton and one electron were involved in the free radical neutralization reaction. The mechanism of the reaction of OA with DPPH can be explained as the oxidation of the hydroxyl group in the 3-position on OA to the ketone group (Figure 11). This mechanism is consistent with observations regarding the OA redox reaction at the electrode, where one electron and one proton are transferred in the oxidation reaction.

The obtained results confirm that OA has strong antioxidant properties, which may be valuable in a pharmacological context, including in the treatment of diseases associated with oxidative damage. The use of the MWCNTs/SPE electrode allowed for quick and precise examination of these properties, which suggests potential applications of this technique in research on other natural antioxidants. Research on the redox kinetics of OA and its antioxidant activity may constitute the basis for further work on the development of drugs using this compound.

Further investigations concerning the antioxidant mechanisms of OA were conducted by Matysik-Woźniak [43]. The results of this research indicated that OA causes inhibition of the activity of pro-oxidant enzymes such as matrix metalloproteinases (MMPs) and nicotinamide adenine dinucleotide phosphate-oxidase (NADPH-oxidase (NOX)). The relationship between antioxidant activity and the abundance of MMPs results from the impact of oxidative stress on the regulation of the expression and activity of these enzymes and from the potential of antioxidants to modulate their action. Metalloproteinases play a key role in the degradation of the extracellular matrix (ECM), and their hyperactivity is associated with many pathological conditions, including cancer and inflammatory, cardiovascular, and neurodegenerative diseases [44]. In turn, the relationship between antioxidant activity and the abundance of NOX results from the role of this enzyme in generating ROS and the action of antioxidants that neutralize ROS and regulate NOX activity. NOX is a key source of ROS in multicellular organisms and its excessive activity leads to oxidative stress, which is associated with many diseases [45].

The potential ability of OA to act against “lipopolysaccharides-induced” oxidative stress (“LPS-induced” oxidative stress) in macrophages is another type of antioxidant activity of the mentioned triterpene [46]. In fact, lipopolysaccharides (LPSs) themselves do not directly produce proinflammatory mediators nor do they cause oxidative stress. De Stefani et al. investigated the activity of OA that was incorporated into two types of microemulsions (MEs) against “LPS-induced” oxidative stress in RAW 264.7 murine macrophages [46]. Through the production of pro-inflammatory mediators, LPSs evoke oxidative stress. Upon LPS stimulation, macrophages reprogram their metabolism by increasing the generation of ROS, which are believed to be involved in the mechanism of LPS-induced cellular toxicity. RAW 264.7 murine macrophages were used as an experimental model to evaluate the effects of OA and its two MEs against LPS-induced cytotoxicity and ROS production. LPSs were used to activate macrophages by triggering a sharp increase in intracellular ROS levels, which generated an oxidative stress condition.

The potential enhancement of the activity of OA against LPS-induced cellular toxicity was evaluated using two different OA-loaded MEs. The researchers demonstrated that carrier-loaded OA had a significant protective effect against both LPS-induced cytotoxicity and intracellular ROS formation. As OA, a plant triterpenoid, is found in many foods and used in traditional medicine to treat various diseases, MEs could be a promising formulation to enhance the oral bioavailability of OA. This could allow for the evaluation of its potential clinical use in treating chronic diseases [46].

## 4. How Is the Antioxidant Activity of OA Expressed: Antioxidant Activity of OA as a Method of Overcoming Other Diseases

### 4.1. Antihypoglycemic/Antidiabetic and Antioxidant Activity of OA

Diabetes is a chronic disease characterized by the body’s inability to properly process blood glucose and regulate its levels, either due to insufficient insulin secretion from the pancreas or impaired insulin action [47]. Somova and co-workers found that OA could prevent the development of insulin-resistant hypertension on the basis of its potent antioxidant and hypoglycemic effects [48]. They investigated the potential use of antioxidant therapy in conjunction with traditional antihypertensive drugs to mitigate the effects of oxidative stress on the blood vessels, the arterial pressure, and low-density lipoproteins.

Hyperglycemia disrupts the prooxidant/antioxidant balance, leading to increased free radicals and reduced antioxidant levels. Free radicals react with lipids, causing peroxidative changes that result in enhanced lipid peroxidation. Various cellular defense mechanisms, including enzymatic and nonenzymatic scavenging systems, regulate the level of lipid peroxidation in cells. Increased free radical production in diabetes has cytotoxic effects on membrane phospholipids, resulting in the formation of toxic products like malondialdehyde (MDA). Enzymes such as superoxide dismutase (SOD) and glutathione peroxidase (GSH-Px) protect cells and tissues from oxidative damage [48].

In their study, Gao and co-workers [49] assessed the antidiabetic and antioxidant effects of OA derived from waxleaf privet leaves (*Ligustrum lucidum* W.T.Aiton; Oleaceae) in alloxan-induced diabetic rats. They based their assessment on measurement of the observed blood glucose levels, lipid profile, liver enzymes (aspartate aminotransferase, AST; alanine aminotransferase, ALT; and alkaline phosphatase, ALP), activity of the antioxidant enzymes (SOD, GSH-Px), and level of MDA in the tissues. The results clearly indicated that OA reduced the observed blood glucose levels both in the short term (2 h after administration) and in the long term (40 days), lowered the levels of total cholesterol, triglycerides, and low-density lipoprotein cholesterol (LDL cholesterol), and increased the high-density lipoprotein cholesterol (HDL cholesterol) value. Moreover, it reduced the level of MDA (which is a product of lipid peroxidation) and increased the activity of SOD and GSH-Px, which indicates an antioxidant effect.

Wang et al. also performed a number of studies on the effect of OA (OA) on hepatic insulin resistance, especially in type 2 diabetes [50]. OA, as determined by the publications cited above, is a natural compound with antioxidant, anti-inflammatory, and hypolipidemic properties, which makes it potentially effective in the treatment of metabolic disorders. Type 2 diabetes is a metabolic disease that is characterized by insulin resistance. Insulin resistance often co-occurs with other health problems, such as obesity, dyslipidemia, hypertension, and cardiovascular diseases [50]. OA may alleviate insulin resistance through its antioxidant effects, reducing inflammation and improving lipid metabolism. An experiment was conducted in which mice with type 2 diabetes were administered OA for two weeks. A significant improvement in their glucose and lipid metabolism was noted, as well as a reduction in the body weight, liver weight, and adipose tissue of the mice. Additionally, OA was observed to protect the liver against damage and improve its functions. It lowered the fasting glucose levels of the mice and improved their glucose and insulin tolerance. It increased insulin signaling and inhibited gluconeogenesis (producing glucose in the liver). OA reduced the production of ROS in the mitochondria, which is one of the major factors that leads to cell damage and the development of insulin resistance. It also increased the GSH levels, which helped maintain the redox balance in the mitochondria and improve their function. OA reduced the levels of inflammatory markers such as interleukin-1 beta (IL-1β), interleukin-6 (IL-6), and tumor necrosis factor alpha (TNF-α), suggesting its ability to alleviate inflammation associated with diabetes and obesity. Therefore, OA may have potential for therapeutic use in the treatment of type 2 diabetes and other metabolic disorders.

OA has broad potential in the treatment of type 2 diabetes thanks to its multidirectional effect on various aspects of the disease, which range from improving insulin secretion to anti-inflammatory and antioxidant effects. In their study on the antidiabetic activity of OA, Castellano et al. showed [51] that, in addition to its numerous antidiabetic effects, OA has an antioxidant effect of increasing the activity of enzymes such as SOD and GSH-Px, which protects pancreatic β cells from damage. The mentioned antidiabetic effects of OA are as follows: OA inhibits carbohydrate-digesting enzymes, i.e., α-glucosidase and α-amylase; increases insulin biosynthesis and secretion; activates muscarinic receptors and the TGR5 receptor (Takeda G protein-coupled receptor 5), which supports the regeneration of beta cells and their functioning; acts on insulin receptors and activates the PI3K/AKT pathway (phosphatidylinositol 3-kinase and activated protein kinase), which improves the sensitivity of cells to insulin and supports the storage of glucose in the form of glycogen; and activates the adenosine monophosphate-activated protein kinase (AMPK) pathway). Moreover, OA can modulate enzymes related to insulin biosynthesis and signaling, and can activate signal transduction pathways such as Nrf2. Activating this pathway results in the expression of genes that are responsible for antioxidant protection and reduces inflammation by inhibiting the NF-kB transcription factor.

### 4.2. Neuroprotective and Antioxidant Activity of OA

Hyperglycemia, a defining characteristic of diabetes, enhances the non-enzymatic glycation reaction (N-EGR), resulting in the formation of advanced glycation end products (AGEPs), which are implicated in the pathogenesis of diabetic complications. This process concurrently generates ROS such as hydroxyl and superoxide anion radicals. The glycation-induced structural modifications of proteins can impair their function, thereby exacerbating oxidative stress and contributing to disease progression in diabetic patients [52]. As Ding and co-workers reported, OA was proven to exhibit significant anti-glycation properties, which may play an important role in mitigating diabetic complications [52]. In particular, OA has demonstrated a clear dose-dependent inhibition of AGE formation. This effect is likely mediated by its ability to scavenge superoxide anion radicals. The presence of these ROS during the N-EGR contributes to the formation of reactive dicarbonyl intermediates, which are key precursors in the development of AGEPs. By reducing ROS levels, OA may limit the accumulation of these intermediates and thereby disrupt the glycation cascade. These findings support the potential therapeutic role of OA as an antioxidant agent in the prevention or attenuation of glycation-related pathologies, particularly hyperglycemic conditions that are associated with diabetes [52]. Researchers have reported that oleanolic acid (OA) almost completely inhibited the formation of AGEPs at a concentration of 500 μg/mL, likely due to a reduction in the levels of AGEPs’ precursors. Moreover, OA demonstrated potent hydroxyl radical (^•^OH) scavenging activity, nearly eliminating ^•^OH at a concentration of 5 μg/mL. It also exhibited strong superoxide anion (^•^O_2_^−^) scavenging capacity, removing approximately 93% of ^•^O_2_^−^ at 50 μg/mL. In addition, Ding et al. observed that the presence of Cu^2^^+^ ions led to a decrease in the absorbance of OA, indicating its capacity to chelate metal ions. These combined actions—free radical scavenging and metal ion chelation—suggest that OA may effectively inhibit protein glycoxidation and thereby offer protective effects against the progression of diabetic complications [52].

Castellano and his group studied the effects of OA on microglia activation, which is associated with neurodegenerative diseases such as Alzheimer’s disease [53]. The activation of microglia leads to the production of harmful inflammatory substances and ROS, which can damage neurons. The results showed that OA significantly reduced the production of inflammatory cytokines (IL-1β, IL-6, TNF-α), which are responsible for inflammation in the brain. This happened by reducing the expression of genes that encode these cytokines. The above triterpene also reduced the production of nitric oxide (NO), which, in excess, leads to neuronal damage. This effect was associated with a reduction in the activity of the inducible nitric oxide synthase (iNOS). OA increased the level of GSH, which is a key antioxidant in cells. This contributed to a reduction in the oxidative stress induced by LPSs. The mechanism of the antioxidant action of OA is exerted by OA modulating key signaling pathways that are related to the antioxidant and inflammatory response, such as the Nrf2 pathway, which activates genes that are responsible for cellular protection against oxidative stress. The mentioned triterpene acid reduces the activation of NFκB, which is the main regulator of the inflammatory response, which leads to a reduction in the level of inflammatory cytokines. OA has potential as a neuroprotective agent that can be used in the treatment of neurodegenerative diseases, such as Alzheimer’s disease, to reduce inflammation and oxidative stress in the brain [53].

Ischemic stroke, caused by limited blood flow to the brain, can lead to permanent damage to nerve cells. The mechanisms that lead to this damage include oxidative stress, inflammation, and apoptosis (cell death). Therefore, research into antioxidants that could reduce the effects of stroke is of great importance. OA is a natural compound that has anti-inflammatory and antioxidant properties. It is commonly used to treat hepatitis in China, but its neuroprotective potential in the context of ischemic stroke has not been previously thoroughly investigated. Rong and co-workers investigated whether OA could protect the brain against damage caused by ischemic stroke and hydrogen peroxide-induced oxidative stress in neuronal cells [54]. Experiments were performed both in vivo (on mice and rats) and in vitro, with the application of PC12 cells derived from a pheochromocytoma tumor on a rat’s adrenal medulla [55]. Oxidative stress was induced in neuronal cells with hydrogen peroxide (H_2_O_2_).

Various parameters were assessed, such as the cell survival, neurological functions, brain necrotic area, brain edema, antioxidant enzyme activities (SOD and GSH-Px), and level of oxidative stress markers (such as MDA). The results revealed that OA increased the activity of antioxidant enzymes such as SOD and GSH-Px and, at the same time, reduced the MDA level, which indicates a reduction in oxidative damage. Thus, OA effectively protected PC12 neuronal cells from hydrogen peroxide-induced damage, improving their survival and reducing lactate dehydrogenase leakage, which indicates reduced cellular toxicity. OA also improved the mitochondrial function in cells by preventing the loss of mitochondrial membrane potential and increasing the activity of succinate dehydrogenase (SDH). To sum up, OA showed strong neuroprotective properties in animal models of stroke and in neuronal cells subjected to oxidative stress. This effect is related to its ability to modulate endogenous antioxidant mechanisms and protect mitochondria. These results suggest that OA may be a promising candidate for treating stroke and other diseases related to oxidative stress [54].

Gu et al. examined the effect of OA on inflammation and apoptosis in a PC12 cell model subjected to oxygen and glucose deprivation and reoxygenation (OGD/R), which represented an in vitro simulation of cerebral ischemia-reperfusion injury (CIRI) [56]. The scientists also investigated the role of the microRNA miR-142-5p as a potential therapeutic target. MicroRNAs are key regulators of gene expression and play essential roles in the pathobiology of cancer, including the initiation and progression of tumors. They typically function via canonical pathways, where mature microRNAs bind to target messenger RNAs (mRNAs), leading to translational repression or mRNAs degradation. miR-142-5p, which is typically downregulated in breast cancer, has been identified as a tumor suppressor. Its aberrant expression contributes to breast cancer development by impairing the regulation of genes involved in cell proliferation and survival [57]. In the studies conducted by Gu and co-workers, PC12 cell models were subjected to OGD/R and treated with OA solutions of various concentrations [56]. The study showed that OA at a concentration of 40 μmol effectively increased the viability of PC12 cells after OGD/R. It reduced inflammatory markers, such as TNF-α, IL-1β, IL-6, and oxidative stress (ROS, MDA), while increasing the level of SOD. OA reduced apoptosis, which was confirmed by a decrease in the expression of the B-cell lymphoma 2-associated (Bcl-2 associated) protein x (Bax) and caspases (cleaved caspase-3, caspase-9) and an increase in the level of Bcl-2; Bcl-2 is a mitochondrial transmembrane protein that regulates the intrinsic apoptosis pathway. The expression of miR-142-5p, elevated in the OGD/R model, was reduced after treatment with OA, indicating a mechanism of action that is dependent on this molecule. In this way, the effectiveness of the mentioned triterpene in reducing inflammation and apoptosis in the CIRI model was confirmed. Based on the obtained results, it can be clearly stated that OA showed a protective effect in the CIRI cell model by inhibiting inflammation and apoptosis, which are related to the regulation of miR-142-5p [56].

The next study proving neuroprotective and antioxidant activity of PA was performed by Ling and co-workers [58]. This study investigated benefits of OA in both cell and animal models of stroke. In human nerve cells exposed to low oxygen and glucose, oleanolic acid reduced cell damage and the buildup of harmful reactive oxygen species by regulating a pathway involving the proteins glycogen synthase kinase-3 beta and heme oxygenase-1. In rats with stroke caused by blocked brain arteries, treatment with oleanolic acid reduced brain damage and improved neurological function. Treated rats also had more healthy nerve cells and fewer dying cells in the affected brain areas. The treatment lowered oxidative stress and supported protective protein activity. These findings suggest that oleanolic acid could be a promising treatment for stroke due to its antioxidant and nerve-protective properties [58].

### 4.3. Cardioprotective and Antioxidant Activity of OA

Doxorubicin (Dox) is an effective anticancer drug, but causes cardiotoxicity, which limits its use. This effect is associated with damage to the heart muscle through oxidative stress, the overproduction of free radicals, calcium overload, and the apoptosis of heart muscle cells. Goyal and co-workers assessed the effects of OA on the hearts of rats exposed to Dox [59]. The study was conducted on Wistar rats, which are albino, outbred laboratory animals that are commonly employed in biomedical and pharmacological research. They are characterized by their relatively long lifespan, high incidence of spontaneous tumor development, and docile temperament, which makes them particularly suitable for studies related to aging, oncology, and metabolic diseases [60]. These rats were divided into four groups. In one group, cardiotoxicity was induced with Dox and then treated with OA and amifostine (a known protective agent). Hemodynamic parameters, cardiac enzymes (such as creatine kinase-myocardial band, CK-MB, and lactate dehydrogenase, LDH), the oxidative stress, and other biochemical indicators were analyzed. Doxorubicin caused an increase in blood pressure, changes in the function of the left ventricle, and an increase in markers of cardiac damage (CK-MB, LDH). Changes in the level of liver enzymes (alkaline phosphatase, ALP; serum glutamic-oxaloacetic transaminase, SGOT; and serum glutamic pyruvic transaminase, SGPT) were also observed. OA showed a strong protective effect that was comparable to that of amifostine. OA lowered the observed blood pressure, improved the left ventricular function, and reduced the level of oxidative stress. OA has been shown to reduce cardiac tissue damage, which was confirmed by histopathological analysis. The protective mechanism of OA is as follows: OA acts by increasing the level of antioxidants such as GSH, CAT, and SOD and decreasing the level of MDA, which confirms its action as an antioxidant and protects the heart against oxidative stress induced by Dox. OA can therefore be used as an adjuvant in therapy with Dox to reduce the negative effects of Dox on the heart. The natural origin of OA makes it a potentially readily available therapeutic agent [59].

### 4.4. Hepatoprotective and Antioxidant Activity of OA

Fu and co-workers conducted research [61] in an animal model (red swamp crayfish, *Procambarus clarkia* Girard; Cambaridae) to evaluate the beneficial effects of OA in a red crayfish culture, focusing on its impact on growth, the antioxidant activity in the hepatopancreas, innate immunity, and the structure of the intestinal microbiota. Crayfish were fed for eight weeks with a diet containing different concentrations of OA (0, 250, 500, 750, and 1000 mg/kg). Growth parameters, digestive and antioxidant enzyme activities, the blood lipid levels, and the structure of the intestinal microbiota were measured, and it was observed that a higher OA concentration (500–1000 mg/kg) was associated with greater weight gain, higher growth efficiency, and a lower feed conversion rate compared to the control group. The levels of AST and ALT enzymes (markers of liver damage) decreased with an increasing amount of OA in the diet, indicating a protective effect of OA on the liver. Antioxidant indices, such as the GSH level and antioxidant enzyme activities, were higher in the OA groups, suggesting better protection against oxidative stress. There were no significant changes in the structure of the intestinal microbiota between groups, suggesting that OA does not significantly affect the composition of intestinal microorganisms [61].

### 4.5. Nephroprotective and Antioxidant Activity of OA

Fibrosis of the renal parenchyma is the final stage of many kidney diseases, such as diabetic nephropathy and glomerulonephritis. The principal cause of this phenomenon is oxidative stress, which leads to inflammation and scarring of the kidneys. Chung and his group conducted a study on mice (eight-week-old male C57BL/6 mice) that underwent unilateral ureteral obstruction (UUO), which is a standard model for inducing renal fibrosis [62]. Mice were treated with OA intraperitoneally one day before UUO, and this treatment was continued for 3 or 7 days after surgery. Then, changes in the kidneys were analyzed in terms of damage, fibrosis, and oxidative stress. The results showed that mice exposed to OA exhibited a significant reduction in the amount of collagen in their kidney tissue and a reduction in their number of inflammatory cells (macrophages). Furthermore, OA increased the Nrf2 activity, which reduced the oxidative stress in the kidney, mainly by increasing the levels of antioxidant enzymes such as heme oxygenase-1 (HO-1) and NADPH—quinone oxidoreductase 1 (NQO1). There was also less lipid peroxidation damage. There were fewer cells that underwent apoptosis in the kidneys of mice treated with OA, suggesting protection against further damage. The probable mechanism of action of this protection is as follows: OA induces Nrf2 by activating signaling pathways that lead to the dissociation of Nrf2 from its cytoplasmic repressor Keap1 (Kelch-like ECH-associated protein 1), allowing Nrf2 to translocate to the nucleus and initiate the transcription of β (ARE)-driven genes). This effect moves to the cell nucleus, where it regulates the expression of genes that are responsible for the antioxidant and anti-apoptotic response. This helps reduce kidney damage caused by oxidative stress and inflammatory processes. The conclusion is that OA has therapeutic potential in the treatment of kidney diseases where oxidative stress plays a key role. By activating antioxidant mechanisms, this acid may reduce kidney damage and slow down the progression of renal fibrosis [62].

Diabetic nephropathy (DN) is the leading cause of end-stage renal disease in diabetic patients, and is related to renal damage induced by oxidative stress and endoplasmic reticulum (ER) stress. Lee conducted a study on the potential therapeutic properties of OA and N-acetylcysteine (NAC) in the treatment of diabetic nephropathy [63]. Diabetes leads to kidney damage by increasing oxidative stress and ER stress, which in turn leads to renal fibrosis and renal failure. In a rat model, the effects of OA and NAC treatment were studied for 20 weeks. In addition to valuable, strictly nephroprotective properties (e.g., reducing albuminuria and improving kidney structure), OA reduced the level of ROS, which led to an increase in the activity of SOD and other antioxidant enzymes in the kidneys. This mechanism of nephroprotective action resulted from the reduction in oxidative stress and ER stress, which inhibited the processes that lead to renal fibrosis and apoptosis (cell death). These compounds also affected the transforming growth factor-β/SMAD 2 and SMAD 3 (TGF-β/SMAD2/3; SMAD means suppressor of mothers against decapentaplegic) signaling pathways, which are associated with the development of fibrosis [63].

Ischemia/reperfusion (I/R)-associated acute kidney injury is a serious clinical problem that often leads to serious health complications. The mechanism of this type of kidney damage is related to three main factors: oxidative stress, inflammation, and apoptosis (cell death). An increase in the ROS level and a weakening of the action of antioxidant enzymes lead to additional damage to kidney cells [64]. Studies on rats conducted by Long et al. showed that the administration of OA before I/R induction significantly reduced the resulting renal damage [64]. This was manifested by a reduction in the levels of creatinine and blood urea nitrogen (BUN), as well as a reduction in markers of kidney damage, such as KIM-1 (kidney injury molecule 1) and LDH. The protective mechanism of OA was expressed in three ways: (1) An antioxidant effect: OA lowers the level of lipid peroxidation products (such as MDA) and increases the activity of antioxidant enzymes such as SOD, CAT, and GSH-Px. OA also increases levels of GSH, a key cellular antioxidant. (2) An anti-inflammatory effect: OA reduces the level of pro-inflammatory cytokines (interferon-γ, IFN-γ; interleukin 6, IL-6) and enzyme myeloperoxidase (MPO), and increases the level of anti-inflammatory interleukin 10 (IL-10). (3) An anti-apoptosis effect: OA reduces the number of cells undergoing apoptosis and lowers the content of caspase-3, a key enzyme in the apoptosis process. OA stabilizes the Nrf2 signaling pathway, which regulates the expression level of glutamate-cysteine ligase catalytic subunit (GCLc)—a key enzyme that is responsible for the synthesis of GSH. The activation of this pathway helps maintain high GSH levels, which protects the kidneys against oxidative damage. The above results indicate that OA may be an effective protective agent in the treatment of renal ischemia-reperfusion-induced damage, mainly due to its antioxidant, anti-inflammatory, and anti-apoptosis properties. Stabilizing the Nrf2 pathway and maintaining GSH levels play a key role in this process [64].

### 4.6. Antiatherogenic and Antioxidant Activity of OA

Atherosclerosis is a common problem in diabetic patients, and mainly results from damage to the endothelial cells of blood vessels caused by oxidative stress. OA, a natural antioxidant, has protective properties against such damage [65]. In research conducted by Zhang et al., the anti-atherosclerotic effect of the mentioned triterpene was assessed on HUVEC cells (human umbilical vein endothelial cells) that were exposed to high glucose concentrations, which was intended to simulate the damage that occurs in diabetes [65]. Damage to endothelial cells by oxidative stress caused by hyperglycemia is a key factor in the development of atherosclerosis [66]. Research by Zhang and co-workers suggests that OA may protect endothelial cells from oxidative damage. First of all, this triterpene improved cell survival by reducing the level of ROS and protecting mitochondria from damage. Moreover, OA restored normal levels of antioxidant enzymes such as SOD and CAT, reduced the level of MDA, an indicator of lipid peroxidation, and reduced apoptosis (cell death) induced by oxidative stress. The anti-atherosclerotic effect of OA results from its ability to inhibit oxidative stress by activating the protein kinase B/endothelial cell nitric oxide synthase (AKT/eNOS) pathway, which promotes the production of nitric oxide (NO), which is crucial for the proper function of vessels and leads to the improved functioning of endothelial cells and a reduced risk of their apoptosis [65].

### 4.7. Dermatoprotective and Antioxidant Activity of OA

Suspended particulate matter (PM), especially PM10 (particulate matter with a diameter of 10 μm or less), is one of the main factors that cause health problems, including respiratory and cardiovascular diseases as well as skin aging [67]. Kim et al. tested the protective effect of OA and broad-leaved privet (*Ligustrum lucidum* Aiton; Oleaceae) extracts on skin aging caused by PM10 particles [68]. PM10 activates the aryl hydrocarbon receptor (AhR) in keratinocytes, leading to the increased expression of inflammation-related genes (e.g., CYP1A1, cytochrome P450, family 1, subfamily A, polypeptide 1). This activation leads to inflammatory processes, the accumulation of autophagosomes, and skin aging. OA and *L. lucidum* extract inhibit AhR activation and reduce CYP1A1 expression. They reduce the levels of pro-inflammatory cytokines, such as TNF-α and IL-6, that are induced by PM10. They inhibit the increase in the MMP-1 levels in fibroblasts that is caused by PM10, preventing collagen degradation and wrinkle formation. Moreover, PM10 causes disruptions in the autophagy process in keratinocytes, i.e., the accumulation of LC3-II (lipidated form of microtubule-associated protein 1 light chain) and p62 (stress-inducible protein), which contributes to skin aging. OA reduces autophagy disruption by reducing the levels of LC3-II and p62 markers. OA has a strong dermatoprotective effect, inhibiting the inflammatory processes, autophagy disruption, and collagen degradation caused by PM10. It may, therefore, be a potential ingredient for use in cosmetics or anti-aging therapies related to air pollution [68].

## 5. Methods for Improving the Antioxidant Activity of OA

OA’s poor water solubility (1.748 µg/L = 3.83 nmol [69]) and low bioavailability limit its pharmacological applications. Advancements in drug delivery systems, particularly nanotechnology, offer solutions to enhance the bioavailability of herbal drugs. Nano-based formulations have improved drug bioavailability and solubility, protect against physicochemical degradation, increase therapeutic activities, enhance stability, and enable sustained delivery. Lipid-based drug delivery systems also show promise in improving the solubility, absorption, and bioavailability of poorly water-soluble drugs. Lipid-based formulations for oral administration range from simple oil solutions to complex mixtures of surfactants, co-surfactants, co-solubilizers, and oil.

### 5.1. Microemulsions

Microemulsions (MEs) are dispersed systems consisting of two immiscible phases: an oil phase and water phase. Such formulation enhances rapid solubilization and absorption in the gastrointestinal tract [46]. To improve the solubility and intestinal permeability of OA, De Stefani and co-workers formulated two different microemulsions (named ME-1 and ME-2) containing OA. ME-1 was based on Capmul^®^ PG-8/NF (propylene glycol monoester of caprylic acid), while ME-2 incorporated isopropyl myristate and black caraway (*Nigella sativa* L.; Ranunculaceae) oil as the oil phase. Tween 20 was used as a surfactant in ME-1, and Cremophor^®^ EL (polyoxyl-ethylated castor oil) and Transcutol^®^ HP (diethylene glycol monoethyl ether) were used as surfactants in ME-2 [46].

A solubility study was carried out to select the components of the MEs [46], and phase diagrams were constructed to determine the extent of their stability and appropriate component ratios. Both MEs effectively solubilized OA without destabilizing the system. ME-2 showed better solubility and permeability due to Cremophor’s^®^ properties as a solubilizer. The developed formulations were physicochemically stable and suitable for oral administration. They remained stable for more than 60 days at 4 °C and 25 °C, with ME-1 being slightly more stable. Both MEs significantly improved the rate and amount of release of OA compared to the free compound, increasing its absorption and bioavailability in aqueous media. The MEs also improved the passive permeation of OA, as evidenced by a parallel artificial membrane permeability assay (PAMPA), particularly in the case of the second microemulsion, which was probably due to the advantageous properties of Cremophor and Transcutol (they are known solubilizing agents, absorption enhancers, and P-gp inhibitors). The PAMPA is an in vitro technique that is used to evaluate the passive permeability of compounds. It measures the diffusion of substances from a donor compartment, across a lipid-infused artificial membrane, into an acceptor compartment. The system typically employs a multi-well microtiter plate for the donor phase, over which a membrane–acceptor assembly is placed, forming a configuration commonly referred to as a “sandwich” [38]. In one study, the permeability of OA was increased due to its increased solubility resulting from the presence of surfactants that were used as stabilizers of the internal phase [46].

### 5.2. Nanoemulsions

Alvarado et al. prepared a nanoemulsion (NE) that contained OA, and this nanoemulsion was characterized by dynamic light scattering transmission electron microscopy [70]. The aim of the study presented by Alvarado and co-workers was to investigate the antioxidant and anticarcinogenic potential of OA and UA isolated from Singapore graveyard flower (*Plumeria obtusa* L.; Apocynaceae) on a B16 murine melanoma cell line. The B16 cells were treated with OA and UA, which were either used in a free form or loaded in a NE system. The antioxidant activity of the OA and UA was determined by the DPPH assay. Their cytotoxic activity was evaluated using the SRB (sulforhodamine-B) method [70]. The OA/UA natural mixture showed a high percentage of DPPH inhibition: 86.06% with UV irraditaion and 85.12% without irradiation. Percentages of inhibition greater than 85% were observed for samples with and without ultraviolet irradiation when they were loaded into the NE system. The natural mixture in the NE system displayed cytotoxic activity from 2.9 µmol, while the free compounds showed activity from 17.4 µmol. The researchers concluded that these pentacyclic triterpenes loaded in a NE system could be considered potential tools that warrant further investigation as anticancer agents [70].

### 5.3. Nanoparticles

#### 5.3.1. Self-Assembly Nanoparticles

Zhou prepared OA nanoparticles (OANPs) using the solvent evaporation method, which is based on the principle of small-molecule self-assembly [71]. The above nanoparticles (OANPs) were obtained using a mixture of OA solution in ethyl acetate and polyvinyl alcohol solution in water, which was stirred and sonicated with ultrasound. X-ray diffraction (XRD), Fourier transform infrared spectroscopy (FTIR), and transmission electron microscopy (TEM) techniques were used to analyze and understand the self-assembly mechanism of OANPs. Western blot analysis was used to investigate the antioxidant stress mechanism of these OANPs.

The OANPs were spherical in shape, with an average diameter of 168 nm. They were investigated for their effects on SAH, a severe brain haemorrhagic disease leading to EBI, which is a major cause of high mortality and severe neurological dysfunction. The OANPs were found to decrease the keap1 protein levels and increase the Nrf2 levels in the cell nucleus, which activates the transcription of antioxidant proteins such as HO1 and NQO1. As a result, they reduce neuronal damage and improve neurological function. Unlike traditional drug delivery systems, the studied OANPs consisted only of OA, serving as both the carrier and drug. Consequently, they exhibit enhanced tissue bioavailability and superior antioxidant stress capabilities [71].

#### 5.3.2. Liposomal Nanoparticles

In 2007, Yin and co-workers prepared liposomes containing OA and UA and tested their antioxidant and anti-glycation properties [72]. The liposomes were prepared using phosphatidylcholine, cholesterol, dicetyl phosphate, and citrate or phosphate buffer, in which the liposomes were suspended. The antioxidant effect of both triterpene acids contained in liposomes was compared with the effect of vitamin E (α-tocopherol). The study showed that these acids inhibited oxidation processes more effectively at higher temperatures (75–100 °C) and at lower pH (2–4) values compared to vitamin E. Moreover, the obtained results showed that OA and UA inhibit the formation of glycation products such as pentosidine and carboxymethyl lysine (CML). These compounds may be useful in the prevention of diseases related to glycation processes, such as diabetes and neurodegenerative diseases. OA proved to be more effective in inhibiting these processes than UA. Both acids have a similar chemical structure but differ in their biological activity, which is related to the difference in the position of one methyl group. It was hypothesized that this difference affects the stability of the molecules and their ability to react. Both compounds can be used as dietary supplements to protect against oxidative and glycation stress. For this reason, they may be useful in the prevention and treatment of chronic diseases such as diabetes, Alzheimer’s, and cardiovascular diseases. Oleanolic acid and UA can be used as preservatives in foods, particularly in low pH (acidic) systems, where they can provide antioxidant protection [72].

Wei and his group presented results for the antioxidant activity of OA prefabricated in a form of liposomal nanoparticles (lipo-OANPs) [73]. Such nanoparticles were prepared using a solution of phospholipids, cholesterol, and OA in a mixture of methanol and chloroform,. Homogenization of this solution was carried out followed by drying of the solution into the form of a film. Next, this film was rehydrated and sonicated. The size of liposomal OA nanoparticles (lipo-OANPs) was assessed using transmission electron microscopy by randomly analyzing about 150 of the liposomes. They had a diameter of 140 nm, a spherical shape, and 79% OA encapsulation efficiency. After 24 days of incubation at 37 °C on a shaker (40 rpm), approximately 54% of the OA was released from the nanoparticles. The antioxidant capacity of OA and its nanoparticles in LX-2 cells (human hepatic stellate cell line) subjected to PM2.5-induced inflammation was assessed by free radical neutralization and H_2_O_2_ scavenging assays. Airborne fine particulate matter was defined as PM2.5, which refers to solid and liquid particles in the air that are less than 2.5 μm in diameter [73]. Wei and co-workers found that 0.05–0.3 mM OA or lipo-OANPs had a significant H_2_O_2_-scavenging capacity in LX-2 cells [73].

Further research on liposomal nanoparticles was conducted by Dwiecki and his group [74]. They analyzed how OA and selected phenolic compounds interact with lipid membranes. The aim of the study was to better understand these interactions and their impact on the antioxidant properties of the tested compounds in model lipid membranes. Studies have shown that OA integrates into the hydrophobic core of the lipid bilayer, causing structural changes in the membrane that lead to increased exposure of the membrane surface to water. At higher concentrations (100 µmol), OA causes a reduction in the size of liposomes, which indicates changes in the organization of phospholipids in the membrane. It was found that the presence of OA and phenolic compounds influenced the size of liposomes. OA reduced their size, as did apigenin and rutin. In the case of apigenin, the size of the liposomes decreased from approximately 275 nm to 140 nm at a concentration of 100 µmol. Similar effects were observed for rutin and ferulic acid. The zeta potential of phosphatidylcholine decreased after the addition of OA and phenolic compounds, indicating the interaction of these compounds with the membrane surface [74].

#### 5.3.3. Nano-OA

Wang and co-workers investigated the effects of nano-OA on metabolic disorders in rats that were fed a high fat and fructose (HFF) diet [75]. Nano-OA is a nanoparticle version of the natural triterpenoid OA, and is known for its valuable pharmacological properties. The creation of a nanoparticle form of OA was intended to improve the bioavailability and effectiveness of the mentioned triterpene in the treatment of insulin resistance and other metabolic disorders caused by a HFF diet. The rats were fed a HFF diet for 12 weeks and then injected with various substances, including OA, nano-OA, and rosiglitazone (a drug used to treat type 2 diabetes). It turned out that, in addition to exhibiting strong antidiabetic properties (e.g., improved insulin sensitivity, protection of the pancreas, and liver), the nano-OA significantly reduced the levels of oxidative stress markers such as MDA and nitric oxide (NO) while increasing the levels of enzyme activity antioxidants (SOD, CAT). The research showed that nano-OA is more effective than the traditional form of OA and rosiglitazone in alleviating metabolic disorders caused by a HFF diet. The nanoparticle form of OA improves its pharmacokinetics, bioavailability, and effectiveness, making it a potential agent for the treatment of obesity and diabetes. The study suggests that nano-OA may be a promising new formulation for the treatment of metabolic diseases associated with obesity and diabetes, especially due to its antioxidant properties and improved insulin sensitivity [75].

### 5.4. Gold(I) Complexes

Liu et al. obtained a series of gold(I) complexes with pentacyclic triterpenes, including betulinic acid (BA), UA, glycyrrhetinic acid (GA), and OA derivatives, and evaluated their biological activities [76]. Such complexes were prepared by the action of alkyne esters of triterpene acids with chloro(triphenylphosphine)gold(I) and Cl-Au-PPh_3_ (correct abbreviation: (Ph_3_P)AuCl or Au(PPh_3_)Cl) in tetrahydrofurane (THF). Gold(I) complexes with pentacyclic triterpenes can exert diverse mechanisms of action against cancer cells, including the inhibition of thioredoxin reductase (TrxR). The thioredoxin system, comprising TrxR, Trx, and NADPH, is an enzymatic system that regulates cellular signaling pathways and proliferation. It is involved in various physiological and pathological processes, including apoptosis, chronic inflammation, and cancer. The Trx system can be oxidized by an abundance of ROS. ROS can activate the proliferation of cancer cells and survival pathways at low levels but cause cell senescence or death at high levels [76].

**Figure 12:**
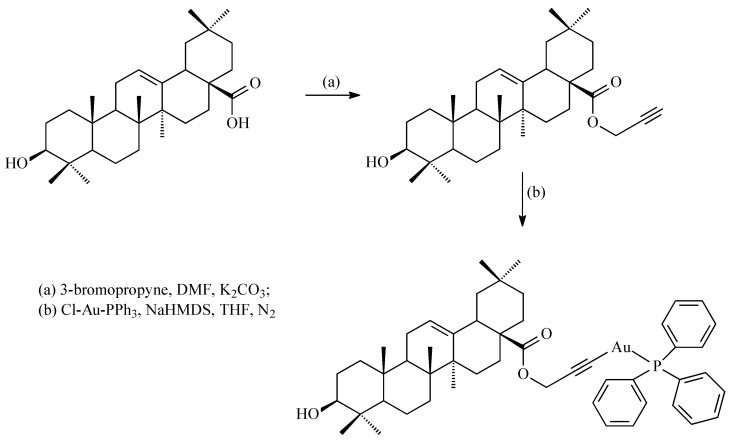
Synthesis of gold–OA complexes. **Legend**: **DMF** = N,N-dimethylformamide; **K_2_CO_3_** = potassium carbonate; **Cl-Au-PPh_3_** = chloro(triphenylphosphine)gold(I); **NaHMDS** = sodium bis(trimethylsilyl)amide; **THF**—tetrahydrofurane; **N_2_** = nitrogen gas. Yield of gold–OA complex: 76% [76].

Gold complexes possess unique electronic structures and active units that participate in redox reactions. These complexes exert antiproliferative effects by interacting with the gold atoms and sulfur donor atoms of enzymes like TrxR. As Liu and his group presented [76], the gold complex of OA (Figure 12) demonstrated a high level of cytotoxicity, specifically against A2780 ovarian cancer cells, with an IC_50_ of 10.24 µmol, which is close to the values for the known anti-cancer agents cisplatine (IC_50_ = 6.49 µmol) and auranofin-Et_3_PAuCl derivative (IC_50_ = 5.74 µmol) [76]. This complex also inhibited the TrxR enzyme, which disrupted the antioxidant defense in cancer cells, increasing their ROS levels. The increase in ROS led to endoplasmic reticulum stress (ERS), disrupting cellular homeostasis and ultimately causing cancer cell apoptosis (programmed cell death). The gold complex also caused mitochondrial dysfunction, contributing to cell death through ROS accumulation.

### 5.5. Nanofibers

Fu et al. prepared nanofibers containing OA (OA nanofibers, OANF) through an electrospinning process and then analyzed the resulting changes in relation to the physicochemical properties of raw OA and its nanofibers to clarify the prepared nanofibers’ improvement in water solubility and skin penetration [77]. The antioxidant and anti-inflammatory properties of newly obtained OA nanofibers (OANFs) were also assessed. Air pollution, including suspended particulate matter (PM), is one of the main factors that causes oxidative stress in the skin. Long-term exposure to PM may lead to skin aging, pigmentation changes, and even inflammation. OA is a natural compound with antioxidant and anti-inflammatory properties, but its limited solubility in water and poor skin absorption limit its use. The developed OANF significantly improved the solubility of OA—by over 1000 times. The electrospinning process reduced the size of the OA particles, increased their active surface area, and changed the crystal structure of OA to amorphous, which allowed for better absorption through the skin. Studies on human keratinocytes showed that OANFs effectively reduced the production of ROS induced by PM. They also reduced the activity of pro-inflammatory proteins such as COX-2 (cyclooxygenase-2, also known as prostaglandin-endoperoxide synthase 2) and NF-κB, as well as proteins associated with skin aging (MMP-1, matrix metalloproteinase 1). OANF showed better antioxidant, anti-inflammatory, and anti-aging effects than OA alone. To sum up, incorporating nanofibers with OA can be an effective way to increase the solubility and absorption of OA, which in turn improves its protective properties against skin exposed to air pollution. These nanofibers effectively reduce the production of ROS and reduce inflammation and aging processes in skin cells, which makes them a promising solution in products that counteract the negative effects of pollution on the skin [77].

## 6. OA Derivatives as Antioxidant Agents

**Figure 13:**
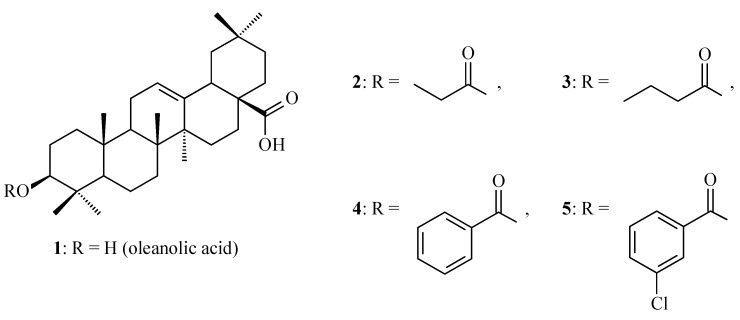
Structures of antioxidant OA derivatives **2**–**5**. Yields of compounds **2**–**5**: **2**: 9.0%, **3**: 7.0%, **4**: 33.0%, **5**: 66.0% [78].

In their experiments, Silva and co-workers esterified three triterpene acids—betulinic acid, UA, and OA—with different anhydrides (propionic, butyric, and benzoic anhydrides) or acyl chloride (3-chlorobenzoyl chloride) [78]. These procedures yielded 12 triterpene acyl derivatives, 4 of which were OA derivatives (compounds **2**–**5**, Figure 13). All these derivatives of three triterpene acids were subjected to a DPPH test, and, as expected, the majority of the compounds were unable to quench the free radical, with 3β-(3-chlorobenzoyl)-OA (**5**, Figure 13) showing a moderate level of antioxidant activity, but a lower level than, for example, quercetin, which is used as a standard.

**Figure 14:**
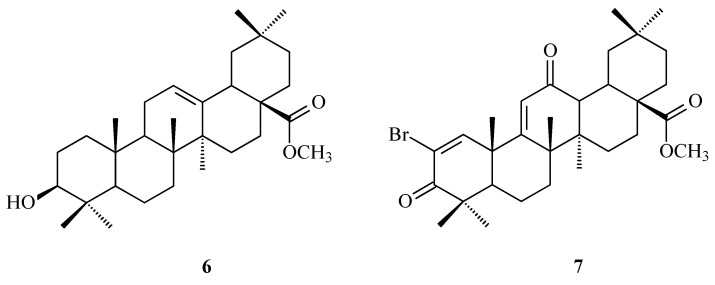
Structures of antioxidant OA derivatives **6** and **7**. Yields of compounds: **6**: 65%, **7**: 35% [79].

To investigate the impact of OA and some of its derivatives on hypertension and oxidative stress, Madlala et al. conducted a study using Dahl salt-sensitive (DSS) and spontaneously hypertensive rats (SHR) as experimental models. In their investigation, the scientists tested a methyl ester of OA (compound **6**, Figure 14) and a brominated derivative of the above methyl ester (compound **7**, Figure 14) [79]. DSS and SHR rats are recognized models for studying hypertension, as they highlight the roles of diet and genetic factors, respectively. The development of hypertension involves abnormal electrolyte handling by the kidneys and endothelial dysfunction caused by oxidative stress. The study examined the effects of OA and its derivatives on the hormone levels and oxidative stress in the liver, heart, and kidney of DSS and SHR rats. Treatment with OA (60 mg/kg^−1^, orally) for 9 weeks significantly reduced the MDA concentration in all tissues and increased the activities of SOD and GSH-Px in the liver and kidney of the rats compared to those of untreated hypertensive animals. The study also aimed to compare the effects of two synthetic OA derivatives with their parent compound [79].

**Figure 15:**
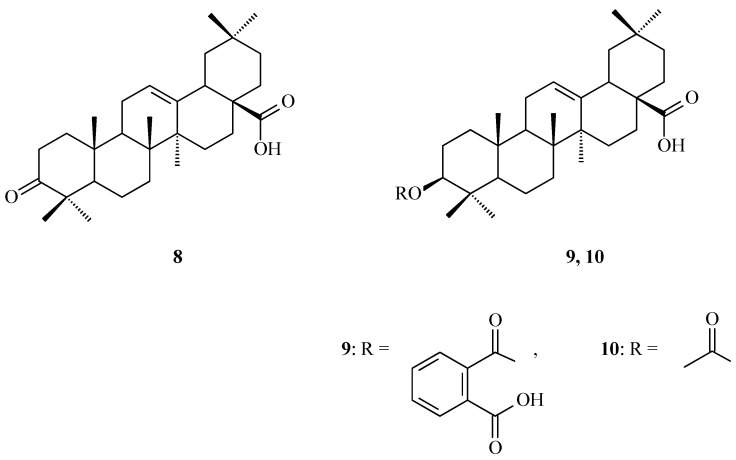
Structures of antioxidant OA derivatives **8**–**10**. Yields of compounds **8**–**10**: **8**: 83.7%, **9**: 94.0%, **10**: 91.2% [27].

To evaluate the antioxidant potential of OA isolated from the dried powdered leaves of *Nepeta leucophylla* Bent. (Lamiaceae) [27] in comparison with its simple derivatives, this triterpene was subjected to a series of chemical modifications. These modifications included acetylation using acetic anhydride in pyridine, acylation with phthalic anhydride in pyridine, and Jones oxidation, and resulted in the formation of derivatives with the structures given in Figure 15 (compounds **8**–**10**): 3-oxo-OA (oleanonic acid, **8**), 3-Pht-OA (3-phthaloyloleanolic acid, **9**), and 3-Ac-OA (3-acetyloleanolic acid, **10**). In this study, DPPH and total antioxidant capacity (TAC) assays were performed to evaluate the antioxidant activity of isolated compounds and synthesized derivatives. As standard positive controls, ascorbic acid and quercetin were applied in these tests. Among the tested compounds, the highest DPPH free radical scavenging potential was exhibited by 3-phthaloyloleanolic acid ((3-Pht-OA), comp. **9**, Figure 15) [27].

The order of the antioxidant potentials (% inhibition in DPPH assay) of triterpenes **1** (Figure 1) and **8**–**10** (Figure 15) was as follows: 3-Pht-OA (**9**; 40.83%) > 3-Ac-OA (**10**; 35.43 66%) > OA (**1**; 23.66%) > 3-oxo-OA (**8**; 15.87%) [27]. The superior DPPH radical scavenging potentials of 3-Pht-OA (**9**) and 3-Ac-OA (**10**) compared to that of OA (**1**, Figure 1) can be attributed to their enhanced solubility and greater propensity to release a proton. This release occurs from the COOH group of the phthaloyl moiety in compound **9** and the acetyl group of 3-Ac-OA in compound **10** (Figure 15), which lead to the formation of an anion stabilized by resonance, a phenomenon that was not observed in OA (**1**). 3-Oxo-OA (**8**, Figure 15) has a lower DPPH radical scavenging potential, which may be related to this compound possessing C-3 oxo functionality, which means that there is a lower probability of the release of proton as compared to OA (**1**), which has a hydroxyl group at the mentioned position.

**Figure 16:**
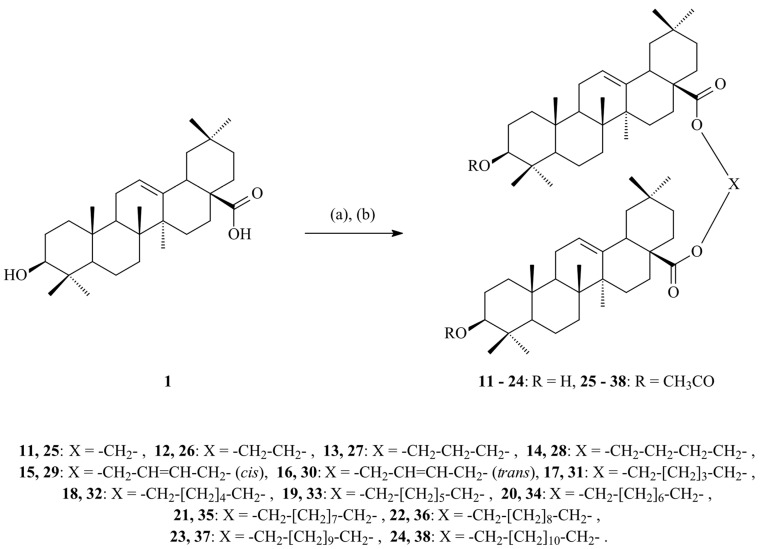
Structures of antioxidant OA derivatives **11**–**38**. **Legend**: (**a**) = N,N-dimethylformamide (DMF), potassium carbonate (K_2_CO_3_), 80 °C; (**b**) DMF, α,ω-dihalogenoalkanes or α,ω-dihalogenoalkenes (Br-X-Br), 80 °C. Yields of compounds **11**–**24**: 92–97% [80]. Yields of compounds **25**–**38**: 90–96% [81].

**Figure 17:**
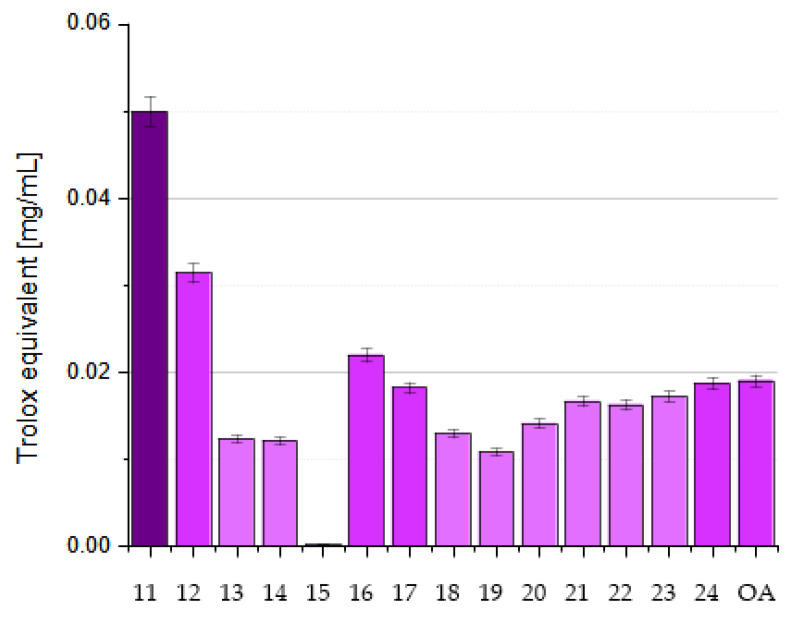
The ability of OADs **11**–**24** and OA to inhibit DPPH radicals, expressed as trolox equivalent [80].

In 2024, in collaboration with other scientists, we published a paper in which we presented the synthesis of a specific group of OA derivatives—dimers—and selected directions of pharmacological activity related to these derivatives [80]. These dimers were obtained by treating the mentioned triterpene (**1**) with α,ω-dihalogenoalkanes or α,ω-dihalogenoalkenes. The obtained dimers, named OA dimers (OADs), consisted of two OA residues that were connected by bridges containing from 1 to 12 carbon atoms, and two of the dimers with four-carbon bridges contained a *cis*- or *trans*-unsaturated bond in their bridge (compounds **11**–**24**, Figure 16). All obtained OADs (**11**–**24**), as well as the parent OA (**1**), were subjected to, among other tests, antioxidant activity testing with the application of the DPPH test. The results were presented as the % inhibition of the DPPH radical and the trolox equivalent (Figure 17) [80].

As shown by the obtained results, most of the dimers exhibited high cytotoxic activity towards all tested cell lines, with IC_50_ values below 10 µmol. Compound **14** showed the highest activity against the SKBR-3 line with an IC_50_ value of 1.12 µmol. The OADs with a one-carbon linker (**11**), those with a two-carbon linker (**12**), and those with a trans-unsaturated four-carbon linker (**16**) showed significantly higher antioxidant activity than OA in the DPPH radical test [80]. The obtained results indicate that OADs may be used as antioxidants, and that they have potential for use in the pharmaceutical industry.

**Figure 18:**
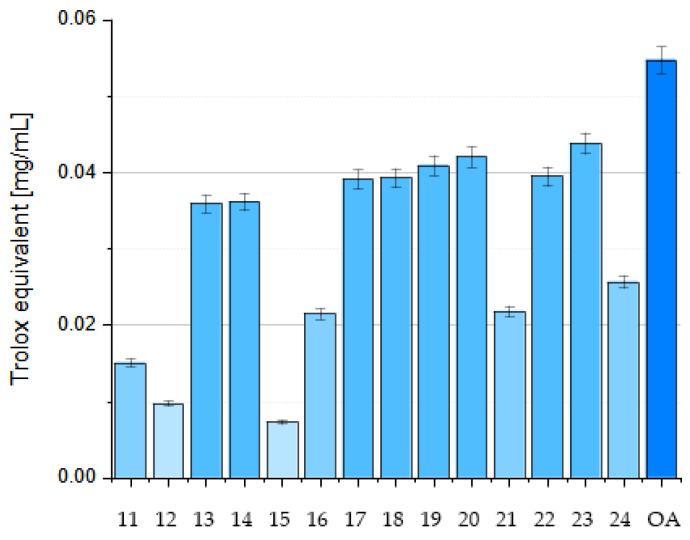
Antioxidant activity in CUPRAC assay of OADs **11**–**24** and OA (**1**), expressed as trolox equivalent [81].

The obtained results, e.g., the cytotoxic activity, selectivity index calculation, and antioxidant activity determined with the DPPH assay, encouraged our group to conduct further research on the obtained OADs [81]. In this work, the obtained OADs (**11**–**24**, Figure 16) were tested on 74 cancer cell lines, including breast, ovarian, prostate, kidney, lung, and nervous system cancer cells. The antioxidant properties of the above compounds were tested using the CUPRAC method (cupric reducing antioxidant capacity assay; presents the ability of tested compound to neutralize radicals), and the results (Figure 18) were compared with results for trolox (a known antioxidant). Most dimers showed clearly greater cytostatic activity than OA alone. The IC_50_ values for many of the cancer cell lines were below 10 µmol, indicating strong anticancer activity. Some dimers, including compounds **17**, **19**, **20**, and **23** (Figure 16), showed higher antioxidant activity compared to OA. The CUPRAC test results indicated the potential of these compounds as effective antioxidants [81]. Once again, it can be stated that OA dimers show promising cytostatic and antioxidant properties that exceed the effects of OA by itself. These compounds may potentially be used as anticancer drugs, especially in the case of breast, prostate, lung, and other types of cancer, and as effective antioxidant agents in the case of numerous diseases whose development is influenced by oxidative stress [81].

**Figure 19:**
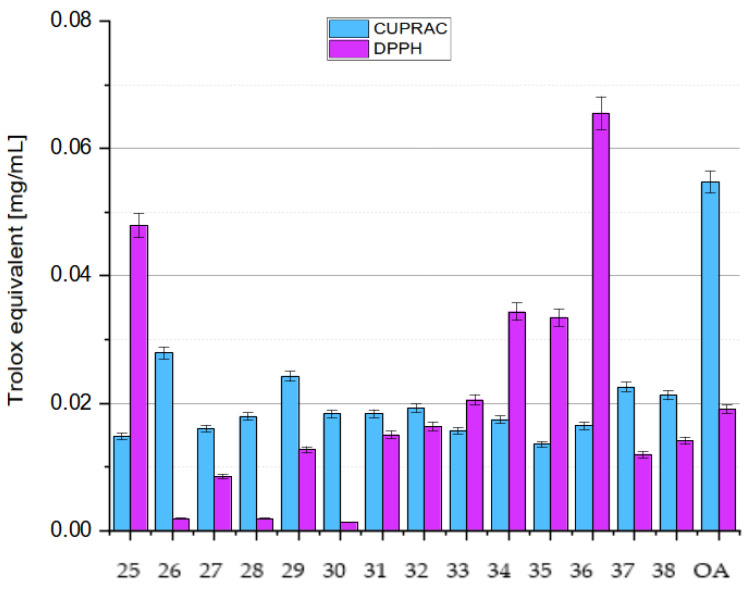
The ability of AcOADs **25**–**38** and OA (**1**) to inhibit the DPPH and CUPRAC radicals (blue bars and pink bars, respectively), expressed as trolox equivalent [82].

As shown by our subsequent publication [82], the acetylation of OA dimers in boiling acetic anhydride induces a simple and very effective chemical transformation that leads to the production of another series of triterpene derivatives—acetylated OA dimers, named AcOADs (compounds **25**–**38**, Figure 16). The new derivatives turned out to be even stronger anticancer agents than their unsubstituted analogues, while DPPH and CUPRAC tests showed significant differences in activity between AcOADs **25**–**38** (Figure 16), as presented in Figure 19.

The differing results between the CUPRAC and DPPH assays highlight the varied antioxidant mechanisms of these compounds. The DPPH and CUPRAC assays measure antioxidant activity through different mechanisms. The DPPH assay evaluates the ability of antioxidants to donate hydrogen atoms to neutralize DPPH radicals, whereas the CUPRAC assay measures the reduction capacity of antioxidants towards the Cu(II)–neocuproine complex. This difference can result in varying antioxidant activity profiles for the same compounds.

The above examples show that the antioxidant activity of OA derivatives is rarely tested. As already mentioned earlier, the etiopathology of many diseases is related to the excessive production of free radicals, in particular ROS. It can therefore be said that the antioxidant activity of various compounds is underestimated, because the results of antioxidant tests and other tests provide not only valuable information about the current directions of pharmacological activity but also constitute valuable tips for examining other potential types of biological activity. These results may also provide valuable guidance on the types of chemical transformations that would be worth carrying out to obtain new derivatives with an even higher level of a given activity.

## 7. Structure–Activity Relationships of Oleanolic Acid and Antioxidant Activity

### 7.1. Stereocenters and Stereochemistry of Oleanolic Acid in Antioxidant Activity

**Figure 20:**
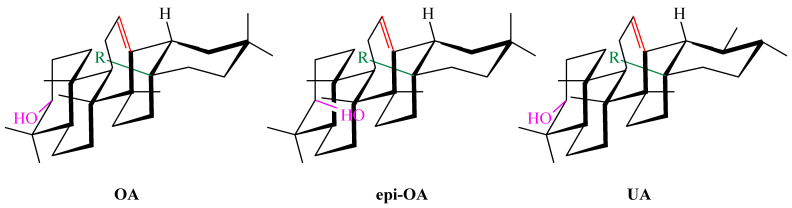
Conformational structures of oleanolic acid (OA), epi-oleanolic acid (epi-OA), and ursolic acid (UA). **Legend**: **R** = COOH.

The OA molecule contains eight stereocenters (chiral carbons) that define its three-dimensional shape. Small changes in its stereochemistry can markedly alter how the molecule interacts with free radicals, metal ions, or biological targets [83]. A prime example is provided by ursolic acid (UA, Figure 6), the C-20 epimer of OA. UA and OA have almost identical structures, differing only in the position of a single methyl group on the E-ring (Figure 1 and Figure 6). This minor stereochemical difference has notable consequences: oleanolic acid tends to exhibit slightly stronger antioxidant effects than ursolic acid in certain models. In leukemic cell assays for H_2_O_2_-induced DNA damage, OA showed significantly higher protective (antioxidant) activity than UA in two cell lines: L1210 (mouse lymphocytic leukemia cell line) and K562 (chronic myelogenous leukemia) [84]. Although UA and OA both significantly prevented oxidative DNA strand breaks at low micromolar concentrations, the methyl placement on their E-rings caused moderate differences in their efficacy, with OA generally being more potent. Consistent with these results, some papers have presented that OA was more effective than UA in inhibiting oxidative glycation products, hypothesizing that the distinct methyl positioning affects the stability and reactivity of the triterpene framework [72]. In one comparison, both acids were found to be able to directly scavenge ROS and prevent damage, but the isomeric variation in UA likely alters the molecule’s conformation or electronic distribution, slightly diminishing its antioxidant reactivity [72]. This illustrates that even a single stereochemical change (here, a shifted methyl stereocenter on ring E, Figure 20) can modulate antioxidant activity.

Beyond the E-ring methyl, the configuration of the C-3 hydroxyl is another stereochemical feature that potentially affects the molecules’ activity. Oleanolic acid naturally occurs with a 3β-OH (axial orientation); although the 3α-OH epimer (3-epi-oleanolic acid) is less common [85], differences in the orientation of this hydroxyl group (Figure 20) could influence the molecule’s hydrogen bonding and how readily the OH can donate a hydrogen atom to quench free radicals. In general, natural pentacyclic triterpenes like OA and UA conserve a β-oriented 3-OH, which appears optimal for their activity [86]. This observation is supported by numerous scientific publications on the pharmacological activity of oleanolic acid derivatives at the C-3 position. While direct antioxidant comparisons of 3-epimers do not exist in the scientific literature, the prevailing 3β configuration may facilitate the molecule’s ability to interact with lipid membranes or enzymes. The stereochemistry at other chiral centers (e.g., ring junctions) enforces the rigid, chair-like conformation of the rings. This rigidity can be important for the functioning of antioxidants: it positions functional groups (like the 3-OH and 17-COOH) in specific spatial orientations. If these stereochemical arrangements are perturbed, the ability of OA to engage in hydrogen transfer or metal chelation might be altered.

### 7.2. Influence of Functional Groups on Antioxidant Activity

The antioxidant capacity of oleanolic acid is intimately tied to the functional groups present on its triterpenoid scaffold. Key functional moieties of OA include: (1) the 3β-hydroxyl group, (2) the 17-carboxyl acid group, and (3) the olefin (double bond) at C-12=C-13. Additionally, various structural modifications (both natural and synthetic), such as the addition of extra hydroxyl groups, the oxidation of functional groups, esterification, or even the dimerization of OA, have been explored to understand their impact on the resulting activity.

#### 7.2.1. Influence of the C-3 Hydroxyl Group on Antioxidant Activity

The presence of a hydroxyl at C-3 is pivotal for the direct free radical scavenging ability of OA [78]. This –OH can act as a hydrogen donor to neutralize radicals, albeit less powerfully than phenolic antioxidants [78]. Removing or modifying this hydroxyl dramatically changes the molecule’s activity. For example, oxidation of OA to replace the 3-OH with a carbonyl (yielding 3-oxo-oleanolic acid, Figure 15) led to a marked drop in its radical-scavenging potency [27]. This suggests that, without the 3-OH, the molecule has a much lower probability of releasing a hydrogen atom to reduce a radical.

Conversely, certain derivatives that retain a proton-donating capability at C-3 can exhibit an enhanced activity. Esterification of the 3-OH—perhaps counterintuitively—can improve a molecule’s antioxidant performance if it introduces a group that readily releases protons and/or improves solubility [78]. The acetylation of oleanolic acid at C-3 (forming 3-Ac-OA, comp. **10**, Figure 15) was found to increase its DPPH scavenging compared to unchanged OA. The 3-Pht-OA (where the 3-OH is esterified with phthalic acid, adding an extra carboxyl group; comp. **9**, Figure 15) was even more potent (~41% quenched).

The enhanced activity of these 3-O-acyl derivatives was attributed to their greater propensity to release a proton and their improved solubility in the assay medium. In 3-Pht-OA (**9**, Figure 15), the introduced phthalate moiety contains an additional −COOH that can donate a proton and form a resonance-stabilized anion, a feature that is not present in the parent compound. Likewise, the acetyl group in 3-acetyl OA may facilitate proton release (for instance, via transient hydrolysis to acetic acid) and improves its lipophilicity, aiding in radical interaction. These findings underscore the importance of the 3-hydroxyl: it serves as a direct antioxidant handle, and modifications at this position can tune the molecule’s hydrogen-donating ability and solubility, thereby modulating its antioxidant capacity [27].

#### 7.2.2. Influence of the C-17 Carboxyl Group on Antioxidant Activity

The C-17 −COOH of oleanolic acid is another critical functional group that influences its antioxidant profile [40]. While the carboxylic proton is not typically a strong hydrogen atom donor in radical scavenging, the carboxylate anion (−COO^−^) that OA can form (especially at a slightly basic or physiological pH) has other antioxidant roles. Notably, the carboxyl group enables the chelation of transition metal ions that catalyze oxidative reactions [87]. OA can bind redox-active metals like iron, thereby inhibiting Fenton chemistry that generates highly reactive hydroxyl radicals [40]. This metal-chelating property is a significant antioxidant mechanism in biological systems, preventing metal-catalyzed ROS formation [88]. The −COOH also increases the polarity, affecting how OA partitions in membranes and aqueous environments. In low-pH (acidic) systems, OA remains protonated and lipophilic.

Modifying the carboxyl group can alter these roles: for instance, methylation of the −COOH to an ester would eliminate the metal-binding ability of OA and could reduce its aqueous solubility, potentially diminishing its antioxidant efficacy despite allowing for a possibly increased cell permeability. On the other hand, introducing additional carboxylate groups (as in the 3-phthaloyl derivative, which adds a second –COOH) significantly boosted the radical scavenging of OA in vitro [27]. This suggests that multivalent acid functionalization can enhance proton donation and electron delocalization after radical capture (the phthaloyl anion is resonance-stabilized).

#### 7.2.3. Influence of the Olefinic Double Bond (C-12=C-13) on Antioxidant Activity

Oleanolic acid’s only unsaturation lies in ring C (between C-12 and C-13). This double bond impacts the molecule’s conformation (Figure 20) and could influence its antioxidant activity in two ways. First, the double bond can participate in radical stabilization through allylic resonance. If a radical forms adjacent to the double bond (for instance, on C-13 after H abstraction), the resulting allylic radical might be somewhat stabilized by conjugation with the C-12=C-13 bond, potentially making OA a more effective chain-breaking antioxidant in lipid peroxidation. However, compared to polyunsaturated antioxidants, this effect in OA is limited. Second, the presence of an olefin can confer slight electrophilicity to the molecule if it is conjugated with a carbonyl. This enone motif is a known Michael acceptor, which can react with thiols of the Keap1 protein, activating Nrf2 and downstream antioxidant enzymes (like heme oxygenase-1) [89]. Oleanolic acid itself lacks a conjugated enone, but it still triggers Nrf2-mediated responses through other mechanisms—possibly through mild oxidative stress signaling or kinase activation [62,65].

The configuration of the double bond (being 12-ene) is also relevant: it defines OA as an oleane-type triterpenoid. If this double bond is hydrogenated (yielding a dihydro-oleanane), the flexibility of the ring increases and the molecule loses any potential allylic stabilization or electrophilic character. While specific studies on a fully saturated oleanolic analog do not exist in the scientific literature, it is expected that saturating the C12=C13 bond might reduce the direct radical-scavenging efficacy slightly, but could improve stability. In contrast, introducing additional unsaturations or electron-withdrawing substituents can enhance antioxidant pathways. Thus, the native olefinic bond in OA contributes subtly to its antioxidant profile by rigidifying its structure and enabling limited conjugation; it also presents a site where functionalization can drastically change the mode of antioxidant action (from purely radical-scavenging to enzyme-inducing, if converted to a Michael acceptor).

#### 7.2.4. Influence of Additional Hydroxyl Group/Groups on Antioxidant Activity

Several naturally occurring OA analogs carry extra functional groups, providing insight into structure–activity relationships. Maslinic acid (2α,3β-dihydroxy-olean-12-en-28-oic acid) is an oleanane triterpene with an extra hydroxyl at C-2. This additional polar group increases the molecule’s hydrophilicity and hydrogen-bonding capacity. Maslinic acid has demonstrated antioxidant effects in various models—for example, it reduced the amount of lipid peroxides in plasma [90] and showed a peroxyl radical scavenging and Cu^2^^+^-chelating ability in vitro [91,92]. Its antioxidant activity is sometimes reported to be comparable to or greater than OA, although the results can be context dependent [92]. The 2α-OH may enable maslinic acid to more effectively chelate transition metals (diols at C-2,3 could form a bidentate chelate with metal ions), and it provides another site for proton donation. Nonetheless, studies on maslinic acid have illustrated that adding hydroxyl groups can enhance the direct ROS scavenging of molecules, up to a point, by increasing the number of hydrogen-donating groups and metal-binding sites. Another example is hederagenin (3β,23-dihydroxy-olean-12-en-28-oic acid), which carries a second hydroxyl on the side chain; this modification is reported to improve the antioxidant capacity in some assays due to providing an additional site for radical attack or metal coordination [93]. In both cases, the presence of extra −OH groups generally strengthens the antioxidant activity of molecules (through direct scavenging and chelation) but can alter their pharmacokinetics.

#### 7.2.5. Influence of Dimerization on Antioxidant Activity

Interestingly, linking two oleanolic acid units can produce dimeric derivatives with superior activity. In our recent research we synthesized several oleanolic acid dimers and found that some exhibited significantly higher antioxidant capacities than monomeric OA [80,81,82]. For instance, certain OA–OA dimers showed enhanced Cu^2^^+^ reducing (CUPRAC) activity, indicating a stronger electron-donating ability [80].

The improved effect of dimerization can be attributed to multiple factors:

(1) Dual radical-binding sites—a dimer essentially doubles the number of functional groups (e.g., two the C-3 hydroxyls) that are capable of quenching radicals or chelating metals;
(2) Increased molecular size—this might localize the dimer in membranes or aqueous environments differently, potentially “shielding” oxidative targets more effectively;
(3) Synergistic stabilization—a radical or electron could delocalize over the two linked OA units in some dimer structures, lowering the overall energy of the radical adduct.

Additionally, dimeric compounds can be designed to incorporate complementary antioxidant mechanisms. For example, an OA dimer linked via a spacer that it can resonate or an OA conjugate attached to a known radical scavenger like catechol could unify multiple modes of action. The observed trend that oleanolic acid dimers outperformed OA in antioxidant assays highlights that structural enlargement and multivalency—while maintaining key functional groups—is a viable strategy to boost the activity of OA [80,81,82]. However, such modifications may also affect the bioavailability and specificity, so they must be balanced carefully.

In summary, the functional groups of oleanolic acid orchestrate its antioxidant behavior through a combination of direct chemical and indirect biological mechanisms. The 3β-hydroxyl and 17-carboxyl are primary sites for direct radical scavenging and metal chelation, respectively, and modifications at these positions strongly impact the antioxidant potential of OA. The olefinic bond at C-12–C-13 contributes to the molecule’s conformation and enables derivatization (e.g., to an enone) that can switch on antioxidant enzyme induction pathways. Additional polar substituents (hydroxyls, sugar moieties in saponins, etc.) generally enhance the molecule’s radical-scavenging capacity (by providing more donating groups or improving solubility) but can alter how it interacts with biological membranes and proteins. Finally, linking oleanolic acid into larger constructs (dimers or hybrids) has shown that multiplying the active pharmacophores yields higher cumulative antioxidant effects. All of these insights underscore a central theme in structure–activity relationships: both the presence and precise configuration of specific groups in oleanolic acid are critical for modulating its antioxidant efficacy.

## 8. Conclusions

To sum up, OA (OA) is a compound with a broad spectrum of antioxidant and anti-inflammatory effects, which is confirmed by numerous in vitro and in vivo studies. The mechanisms of its antioxidant activity include:

1. The deactivation of free radicals: OA can directly neutralize ROS and RNS, such as the hydroxyl radical (^•^OH), the superoxide anion radical (O_2_^−•^), and singlet oxygen (^1^O_2_ or 1ΔgO_2_). In addition, it acts as an electron or proton donor, which allows the transformation of free radicals into less reactive forms [5,23,25,29,31,32,37,41,43];
2. The induction of antioxidant enzymes: OA triggers the activation of transcription factors, specifically Nrf2, which in turn regulate the expression of antioxidant enzymes, including SOD, CAT, and GSH-Px. Increased activity of these enzymes helps neutralize ROS and prevent oxidative damage [37,48,51,52,54,56,59,63,64,65,79];
3. The protection of cell membranes: OA stabilizes lipid membranes, protecting them against lipid peroxidation induced by ROS. This reduces the formation of MDA and other markers of lipid peroxidation [37,49,54,56,59,62,64,65,79];
4. The impact on redox balance: OA supports the regeneration of endogenous antioxidants such as GSH and ascorbic acid (vitamin C) and protects antioxidants against oxidative degradation [37,50,53,59,61,64];
5. Inhibition of the activity of pro-oxidant enzymes: OA can inhibit the activity of ROS-generating enzymes such as NADPH-oxidase. It also acts as an inhibitor of certain MMPs, which generate reactive compounds during inflammatory processes [43];
6. The inhibition of pro-inflammatory pathways: OA inhibits pro-inflammatory pathways such as NF-κB and MAPK, which are associated with the generation of ROS in inflammatory processes [37];
7. The chelation of transition metal ions: OA binds metal ions such as iron, which catalyze Fenton reactions, leading to the generation of ROS [40].

Thanks to these multi-faceted mechanisms, OA has significant antioxidant activity and can protect the body against oxidative stress and related diseases, such as cancer, neurodegenerative diseases, and cardiovascular diseases.

Various structural modifications of OA, both chemical and physical, including dimerization and encapsulation into nanoparticles, showed increased antioxidant and cytotoxic activity against cancer cells. Research results also suggest that OA may be used in the treatment and prevention of diseases related to diabetes and cardiovascular and neurodegenerative diseases. The use of nanotechnology, including nanoparticles and liposomes, increases the bioavailability of OA, which makes it a promising candidate for further research on the treatment of chronic diseases.

Ultimately, OA is a potentially valuable compound with therapeutic properties, but further research is required to fully understand its mechanisms of action and potential for clinical application.

Data quality control is essential in scientific research because it ensures the accuracy, consistency, and reliability of data, which directly impacts the validity of the conclusions of studies. Without proper quality control, data may contain errors, inconsistencies, or biases that can lead to misleading results, faulty interpretations, or irreproducible outcomes. The results of the research on the antioxidant activity of oleanolic acid presented in this paper are very promising and prospective; however, some of them are potentially non-reliable (e.g., the one that found that OA has 88.30 ± 1.84% DPPH-scavenging activity, or that OA presents 97.3% antioxidant capacity). However, OA has emerged as a promising and prospective antioxidant agent due to its strong free radical-scavenging properties and ability to modulate oxidative stress-related pathways. Beyond its molecular interactions, oleanolic acid has shown protective effects in various oxidative stress-related conditions, including neurodegenerative diseases, cardiovascular disorders, liver injury, and cancer. Its low toxicity, widespread natural occurrence, and favorable pharmacological profile make it a highly attractive candidate for therapeutic development. Moreover, ongoing research into oleanolic acid derivatives aims to enhance its bioavailability and potency, further supporting its potential as a novel antioxidant-based therapeutic agent.

## 9. Future Directions

Future research on OA should focus on several key areas that may increase its therapeutic effectiveness and practical application.

The first is the optimization of the bioavailability of OA through the development of advanced delivery systems such as nanotechnologies. OA nanoparticles and liposomal forms significantly increase the stability, solubility, and therapeutic effectiveness of OA, which opens new possibilities in the treatment of metabolic diseases such as obesity and diabetes, as well as in cancer therapy.

Further research should focus on understanding the molecular mechanisms of OA’s action, especially regarding its ability to modulate signaling pathways involved in oxidative stress and inflammation. Particularly promising are studies on the interactions of OA with signaling proteins such as Nrf2, which regulate the expression of antioxidant genes.

Another important direction is structural modifications of OA, which can lead to the creation of derivatives with increased biological activity, including stronger antioxidant and anticancer properties.

Finally, further clinical research on the safety and effectiveness of OA is necessary to evaluate its potential use in the treatment of chronic and degenerative diseases, including cancer, diabetes, and neurodegenerative disorders.

## Figures and Tables

**Table 1:** OA content in the plants mentioned in [5,21,22,23,24,25,26,27,28,29,30,31,32,33,34,35,36]. The OA content was converted to mg/g (or g/mL) of plant material (or plant extract) and expressed as a percentage.

OA Sources and Its Content					
Common Name	Latin Name	Part of the Plant	Content by Weight	Content by %	Reference
wine grapes	*Vitis vinifera* L.	fruits	8.7 mg/g dry fruits	0.87%	[21]
wine grapes	*Vitis vinifera* L.	fruits	n.d. for pure compound	- - -	[22]
olive	*Olea europaea* L.	fruits	n.d. for pure compound	- - -	[22]
Kiyomi oranges	*Citrus reticulata* Blanco × *Citrus* × *aurantium* L.	fruits	110 mg/g dried fruit pomace	11.0%	[23]
mango	*Mangifera indica* L.	peels	0.003 mg/g	0.0003%	[23]
apple	*Malus domestica* (Suckow) Borkh.	peels	0.00011 g/mL	0.011%	[25]
apple	*Malus domestica* (Suckow) Borkh.	peels	3.6 mg/g dry peel weight	0.36%	[26]
white leaved catmint	*Nepeta leucophylla* Benth.	herb	1.1 mg/g dry herb	0.11%	[27]
mastic tree	*Pistacia lentiscus* L.	resin	n.d. for pure compound	- - -	[28]
Indian frankincense	*Boswellia serrata* Roxb. Ex Colebr.	resin	n.d. for pure compound	- - -	[28]
common myrrh	*Commiphora myrrh* Nees	resin	n.d. for pure compound	- - -	[28]
wine grapes	*Vitis vinifera* L.	whole fruits	0.178 mg/g	0.018%	[29]
wine grapes	*Vitis vinifera* L.	fruit peels	0.351 mg/g	0.035%	[29]
wine grapes	*Vitis vinifera* L.	seeds	0.042 mg/g	0.004%	[29]
tiny flower hibiscus	*Hibiscus micranthus* L.f.	herb	up to 3.87 mg/g dry petroleum eter extract	0.39%	[30]
hibiscus Roselle	*Hibiscus deflersii* Schweinf. ex Cufod.	herb	up to 0.41 mg/g dry petroleum eter extract	0.04%	[30]
lemonyellow rosemallow	*Hibiscus calyphyllus* Cav.	herb	up to 1.21 mg/g dry petroleum eter extract	0.12%	[30]
black plum	*Vitex doniana* Sweet	fruits	90.24 mg/g methanolic extract	0.02%	[31]
common olive	*Olea europaea* L.	fruits	5.2 mg/g dry extract	0.52%	[5]
mugwort, african wormwood	*Artemisia afra* Jacq. ex. Willd.	herb	n.d. for pure compound	- - -	[32]
horsewood tree	*Clausena anisata* (Willd.) Hook.f. ex. Benth	herb	n.d. for pure compound	- - -	[32]
dikbas, South African wild pear	*Dombeya rotundifolia* (Hochst.) Planch.	herb	n.d. for pure compound	- - -	[32]
morula, cider tree	*Sclerocarya birrea* (A.Rich.) Hochst.	herb	n.d. for pure compound	- - -	[32]
red currant tree	*Searsia chirindensis* (Baker f.) Moffett	herb	n.d. for pure compound	- - -	[32]
pepper-bark tree	*Warburgia salutaris* (Bertol. f.) Chiov.	herb	n.d. for pure compound	- - -	[32]
lemon balm	*Melissa officinalis* L.	leaves	3.5 mg/g raw leaves	0.35%	[33]
henna tree	*Lawsonia inermis* L.	seeds	n.d. for pure compound	- - -	[34]
Chinese jujube	*Ziziphus jujuba* Mill.	fruits	up to 0.308 mg/g	0.031%	[35]
grape-scented sage	*Salvia melissiflora* Benth.	aerial parts	n.d. for pure compound	- - -	[36]

**Legend**: **OA** = oleanolic acid; **n.d.** = no data.

**Table 2:** Antioxidant activity of OA mentioned in [5,21,22,23,24,25,26,27,28,29,30,31,32,33,34,35,36], as determined with the application of various methods.

OA Antioxidant Activity		
Assay	Results	Reference
DPPH free radical scavenging activity	IC_50_ = 61.5 µg/mL	[21]
DPPH free radical scavenging rate	88.30% inhibition	[21]
DPPH free radical scavenging rate	2.7% inhibition	[24]
ABTS free radical scavenging rate	11.0% inhibition	[24]
FRAP free radical reducing rate	1.2 µmol TE/kg DMP	[24]
DPPH free radical scavenging activity	n.d. for pure compound	[25]
DPPH free radical scavenging activity	23.66% inhibition	[27]
total antioxidant capacity	16.93 mg AAE/g of DPPH	[27]
increase of peroxide value	n.d. for pure compound	[28]
DPPH free radical scavenging activity	n.d. for pure compound	[29]
FRAP free radical reducing activity	n.d. for pure compound	[29]
DPPH free radical scavenging activity	n.d. for pure compound	[30]
DPPH free radical scavenging activity	IC_50_ = 2.80 µg/mL	[31]
DPPH free radical scavenging activity	18.2% inhibition	[5]
FRAP total antioxidant capacity	240.9 µmol	[5]
DPPH free radical scavenging activity	IC_50_ = 32.20 µg/mL	[32]
DPPH free radical scavenging activity	n.d. for pure compound	[33]
DPPH free radical scavenging activity	n.d. for pure compound	[34]
DPPH free radical scavenging activity	n.d. for pure compound	[35]
ABTS free radical scavenging rate	n.d. for pure compound	[35]
ORAC free radical absorbance activity	n.d. for pure compound	[36]
AAPH free radical scavenging activity	n.d. for pure compound	[36]

**Legend**: OA = oleanolic acid; **DPPH** = 2,2-diphenyl-1-picrylhydrazyl radicals assay; **TE** = trolox equivalent; **ABTS** = 2,2′-azinobis(3-ethylbenzothiaziline-6-sulfonate assay; **FRAP** = ferric reducing antioxidant power; **AAE** = ascorbic acid equivalent; **DMP** = dry mango peel; **IC_50_** = half maximal inhibitory concentration; **n.d.** = no data; **ORAC** = oxygen radical absorbance capacity; **AAPH** = (2,2′-azobis(2-amidinopropane) dihydrochloride) assay.

## Abbreviations

The following abbreviations are used in this manuscript (abbreviations are listed in alphabetical order)

−COO^−^	carboxylate anion
−COOH	carboxyl group
−OH	hydroxyl group
°C	degrees Celsius
µg/mL	microgram per milliliter (10^−6^ g per 10^−3^ L)
µg/L	microgram per liter (10^−6^ g per liter)
µmol	micromole (10^−6^ moles)
1Δg O_2_	singlet oxygen
^1^O_2_	singlet oxygen
11-oxo-12-ene	a fragment of an oleanane molecule with a ketone group at C-11 and an unsaturated bond starting at C-12 atom
17-COOH	carboxyl group located at the C-17 position of oleanane skeleton
2α-OH	hydroxyl group located at the C-2 position of oleanane skeleton and at the α orientation
3α-OH	hydroxyl group located at the C-3 position of oleanane skeleton and at the α orientation
3β-OH	hydroxyl group located at the C-3 position of oleanane skeleton and at the β orientation
3-Ac-OA	3-acetyloleanolic acid
3-oxo-OA	oleanonic acid, “3-oxooleanolic” acid (incorrect name)
3-OH	hydroxyl group located at the C-3 position of oleanane skeleton
3-Pht-OA	3-phthaloyloleanolic acid
A2780	ovarian cancer cells
AAE	Ascorbic Acid Equivalent
AAPH	(2,2′-azobis(2-amidinopropane) dihydrochloride)
ABTS	2,2′-azino-bis-3-ethylbenzthiazoline-6-sulfonic acid
AcOADs	acetylated oleanolic acid dimers
AGEPs	advanced glycation end products
A-H	electron donor or proton donor
AhR	aryl hydrocarbon receptor
AKT/eNOS	protein kinase B/endothelial cell nitric oxide synthase
ALP	alkaline phosphatase
ALT	alanine aminotransferase
AMPK	adenosine monophosphate-activated protein kinase
ARE	antioxidant response element
B16	murine melanoma cell line
BA	betulinic acid
Bax	Bcl-2-associated protein x, B-cell lymphoma 2-associated protein x
BHT	butylated hydroxytoluene
Br-X-Br	α,ω-dihalogenoalkane or α,ω-dihalogenoalkene
BUN	blood urea nitrogen
C-12=C-13	double bond between carbon atoms at the 12 and 13 positions of oleanane skeleton
C-2,3	arrangement of two identical elements at the C-2 and C-3 position of the oleanane molecule
C-3 (C-5, etc.)	carbon atom at the position number 3 (number 5, etc.) of oleanane skeleton
C57BL/6	common inbred strain of laboratory mouse
CAT	catalase
CCl_4_/NADPH	carbon tetrachloride/nicotinamide adenine dinucleotide phosphate
CHP	cumine hydroperoxide (correct name: cumene hydroperoxide)
CIRI	cerebral ischemia-reperfusion injury
CK-MB	creatine kinase-myocardial band
Cl-Au-PPh_3_	chloro(triphenylphosphine)gold(I); correct abbreviation: (Ph_3_P)AuCl or Au(PPh_3_)Cl)
Co(II)/EDTA	cobalt(II) ions and ethylenediaminetetraacetic acid
COX-2	Cyclooxygenase-2; prostaglandin-endoperoxide synthase 2
Cu^2+^	element copper in its +2 oxidation state
CYO1A1	cytochrome P450, family 1, subfamily A, polypeptide 1
DMAPP	dimethylallyl pyrophosphate
CML	carboxymethyl lysine
DMF	N,N-dimethylformamide
DN	Diabetic nephropathy
DNA	deoxyribonucleic acid
Dox	doxorubicin
DPPH	1,1-diphenyl-2-picrylhydrazyl
DPPH^•^	1,1-diphenyl-2-picrylhydrazyl radical
DSS	Dahl-salt sensitive (rats)
e^−^	electron, electron donor
ECM	extracellular matrix
epi-OA	epi-oleanolic acid
e.g.,	*exempli gratia* (Lat.), for example (Eng.)
ER	endoplasmic reticulum
ERK	extracellular signal-regulated kinase
ERS	endoplasmic reticulum stress
et al.	*et alia* (Lat.), and others (Eng.)
etc.	*et cetera* (Lat.), and so on (Eng.)
Fe^2+^	element iron in its +2 oxidation state
Fe^3+^	element iron in its +3 oxidation state
[Fe(TPTZ)_2_]^3+^	ferric-tripyridyltriazine
[Fe(TPTZ)_2_]^3+^	reduced ferric-tripyridyltriazine
FRAP	Ferric Reducing Antioxidant Power
FTIR	Fourier transform infrared spectroscopy
CGLc	glutamate-cysteine ligase catalytic subunit
GA	glycyrrhetinic acid
GS	Granny Smith (apple variety)
GSH	glutathione in its reduced form
GSH/GSSG	reduced to oxidized glutathione
GSH-Px	glutathione peroxidase
H_2_O_2_	hydrogen peroxide
HCT-116	colon cancer cell line
HDL	high-density lipoprotein
HFF	high fat and fructose
HO-1	heme oxygenase-1
HPLC	High-Performance Liquid Chromatography
HUVEC	human umbilical vein endothelial cells
IC_50_	minimum inhibitory concentration
i.e.,	*id est* (Lat.), that is, it means (Eng.)
IFN-γ	interferon-γ
IL-1β	interleukin-1 beta
IL-6	interleukin-6
IL-10	interleukin 10
iNOS	inducible nitric oxide synthase
IPP	isopentenyl pyrophosphate
I/R	ischemia/reperfusion
JAK-STAT	Janus kinase/signal transducers and activators of transcription
JNK	cellular Jun N-terminal kinase
K_2_CO_3_	potassium carbonate
K562	chronic myelogenous leukemia
Keap1	Kelch-like ECH-associated protein 1
KIM-1	kidney injury molecule 1
L1210	mouse lymphocytic leukemia cell line
LC3-II	lipidated form of microtubule-associated protein 1 light chain
LDH	lactate dehydrogenase
LDL	low density lipoprotein
Lipo-OANPs	oleanolic acid loaded liposomal nanoparticles
LPS	lipopolysaccharides
LX-2	human hepatic stellate cell line
MAPK	mitogen-activated protein kinase, MAP-kinase
MCF-10A	human breast epithelial cell line
MDA	malondialdehyde
MDA-MB-231	triple negative human epithelial breast cancer cells
MEP	methylerythritol phosphate
MEs	microemulsions
ME-1, ME-2	microemulsion no. 1, microemulsion no. 2
mg	milligram (10^−3^ g)
mg/g	milligram per gram (10^−3^ g per gram)
mg/kg	milligram per kilogram (10^−3^ g per 10^3^ g)
MPO	myeloperoxidase
miRNAs	key regulators of gene expression, play essential roles in the pathobiology of cancer
mmol	millimole (10^−3^ mole)
MMP1	matrix metalloproteinase 1
MMPs	matrix metalloproteinases
MPE	mango peel extract
MVA	mevalonic acid
MWCNTs/SPE	screen-printed electrode modified with multi-walled carbon nanotubes
N_2_	nitrogen gas
NAC	N-acetylcysteine
NADPH	nicotinamide adenine dinucleotide phosphate
NaHMDS	sodium bis(trimethylsilyl)amide
nano-OA	nanoparticles of oleanolic acid
NBT	nitroblue tetrazolium salt
n.d.	no data
N-EGR	non-enzymatic glycation reaction
NF-κB	nuclear factor kappa B
nm	nanometer (10^−9^ m)
nM	nanomole (10^−9^ moles)
NE	nanoemulsion
NO	nitric oxide
NOX	NADPH-oxidase
Nrf2	nuclear erythroid 2-related transcription factor
NQO1	quinone oxidoreductase
^•^O_2_^−^	superoxide radical
^•^OH	hydroxyl radical
OA	oleanolic acid
OADs	oleanolic acid dimers
OANF	oleanolic acid nanofibers
OANPs	oleanolic acid nanoparticles
ODG/R	oxygen and glucose deprivation and reoxygenation
ORAC	Oxygen Radical Absorbance Capacity
P12	pheochromocytoma tumor of the rat adrenal medulla
p62	stress-inducible protein
PAMPA	parallel artificial membrane permeability assay
pH	the negative decadic logarithm of the proton activity in the solution under scrutiny
PI3K/AKT	phosphatidylinositol 3-kinase and activated protein kinase
pK_a_	the negative decadic logarithm of acid dissociation constant
PM	particulate matter
PM2	particulate matter with a diameter of 2.5 μm or less
PM10	particulate matter with a diameter of 10 μm or less
PRX-1	peroxidoxin 1
QZG	human fetal liver cell line
RAW 264.7	murine macrophages
RD	Red Delicious (apple variety)
RG	Royal Gala (apple variety)
ROS	reactive oxygen species
rpm	revolutions per minute
RSA	radical scavenging sctivity
SDH	succinate dehydrogenase
SGOT	serum glutamic-oxaloacetic transaminase
SGP	serum glutamic pyruvic transaminase
SHR	spontaneously hypertensive rats
SMAD	Suppressor of Mothers Against Decapentaplegic
SOD	superoxide dismutase
SRB	sulforhodamine-B
tBHP	tert-butyl hydroperoxide
TAC	total antioxidant capacity
TE	trolox equivalent
TEM	transmission electron microscopy
TGF-β/SMAD2/3	transforming growth factor-β/SMAD 2 and SMAD 3
TGR5	Takeda G protein-coupled receptor 5
THF	tetrahydrofurane
TNF-α	tumor necrosis factor alpha
Trx	thioredoxin
TrxR	thioredoxin reductase
UA	ursolic acid
UUO	unilateral ureteral obstruction
XRD	X-ray diffraction
